# Metal(loid) tolerance, accumulation, and phytoremediation potential of wetland macrophytes for multi-metal(loid)s polluted water

**DOI:** 10.1007/s11356-024-35519-5

**Published:** 2024-11-27

**Authors:** Aqib Hassan Ali Khan, Blanca Velasco-Arroyo, Carlos Rad, Sandra Curiel-Alegre, Carlos Rumbo, Herwig de Wilde, Alfredo Pérez-de-Mora, Sonia Martel-Martín, Rocío Barros

**Affiliations:** 1 International Research Center in Critical Raw Materials for Advanced Industrial Technologies (ICCRAM), University of Burgos, Centro de I+D+I, Plaza Misael Bañuelos s/n., 09001 Burgos, Spain; 2 Department of Biotechnology and Food Science, University of Burgos, Plaza Misael Bañuelos, s/n., 09001 Burgos, Spain; 3 Research Group in Composting (UBUCOMP), Faculty of Sciences, University of Burgos, Plaza Misael Bañuelos s/n, 09001 Burgos, Spain; 4 Department of Soil and Groundwater, TAUW België nv, Waaslandlaan 8A3, 9160 Lokeren, Belgium; 5 Department of Soil and Groundwater, TAUW GmbH, Landsberger Str. 290, Munich, 80687 Germany

**Keywords:** Macrophytes, Constructed wetlands, Heavy metals, Groundwater, Phytostabilization

## Abstract

Natural based solutions, notably constructed/artificial wetland treatment systems, rely heavily on identification and use of macrophytes with the ability to tolerate multiple contaminants and grow for an extended period to reduce contamination. The potential to tolerate and remediate metal(loid) contaminated groundwater from an industrial site located in Flanders (Belgium) was assessed for 10 wetland macrophytes (including *Carex riparia* Curtis, *Cyperus longus* Baker, *Cyperus rotundus* L., *Iris pseudacorus* L., *Juncus effusus* L., *Lythrum salicaria* L., *Mentha aquatica* L., *Phragmites australis* Trin. ex Steud., *Scirpus holoschoenus* L., and *Typha angustifolia* L.). The experiment was conducted under static conditions, where plants were exposed to polluted acidic (pH ~ 4) water, having high level of metal(loid)s for 15 days. Plant biomass, morphology, and metal uptake by roots and shoots were analysed every 5 days for all species. *Typha angustifolia* and *Scirpus holoschoenus* produced ~ 3 and ~ 1.1 times more dried biomass than the controls, respectively. For *S. holoschoenus*, *P. australis*, and *T. angustifolia*, no apparent morphological stress symptoms were observed, and plant heights were similar between control and plants exposed to polluted groundwater. Higher concentrations of all metal(loid)s were detected in the roots indicating a potential for phytostabilization of metal(loid)s below the water column. For *J. effusus* and *T. angustifolia*, Cd, Ni, and Zn accumulation was observed higher in the shoots. *S. holoschoenus*, *P. australis*, and *T. angustifolia* are proposed for restoration and phytostabilization strategies in natural and/or constructed wetland and aquatic ecosystems affected by metal(loid) inputs.

**Graphical abstract:**

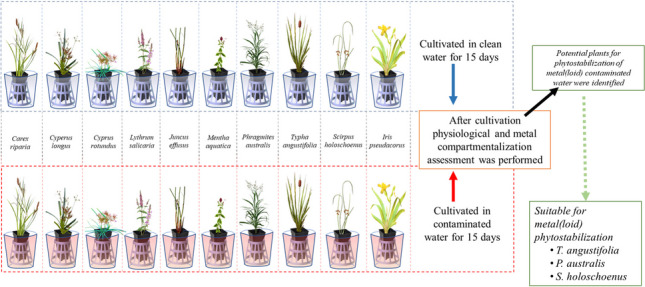

**Supplementary Information:**

The online version contains supplementary material available at 10.1007/s11356-024-35519-5.

## Introduction

Numerous contaminants are released regularly into the environment because of industrial and anthropogenic activities (Curiel-Alegre et al. 2022; Manzoor et al. 2016). Among these, metal(loid)s, commonly named as heavy metals (HMs), are of significant concern as they are indestructible and represent an environmental health hazard, even at low concentrations (Khan and Barros 2023). In addition, metal(loid)s can bioaccumulate and biomagnify via the food chain (Iqbal et al. 2020). Ecotoxicological risk can be further exacerbated, if multiple metal(loid)s are present in the environmental matrix, as it occurs for most metal(loid) contaminated environments (Khan et al. 2019a). Metal(loid)s combinations in environmental matrix can have synergistic (stronger) or antagonistic (weaker) toxicity, mobility, and bioavailability, due to shared mechanisms, altered uptake, or competition (Zhai et al. 2023). Understanding these interactions is crucial for quantifying the capacity for environmental remediation and toxic effects (Moukadiri et al. 2024). Due to their persistent and toxic character, the remediation of metal(loid) contaminated environments is of paramount importance and generally strictly regulated (Lee and Kim 2019; Mushtaq et al. 2020). Aquatic systems are particularly challenging as metal(loid)s can disperse with the (ground)water flow reaching areas far away from the original source. In addition, the redox and chemical form (e.g., in solution, organically bound, etc.) of metal(loid)s can change in the sediment–water interphase affecting their toxicity and mobility (Li et al. 2020).

Contaminant(s) removal using plants, from the aqueous phase (water column or soil/sediment solution), generally occurs via extraction and subsequent accumulation in the shoots or roots or via precipitation in the rhizosphere, hence phytoremediation approaches require treatability testing and optimization prior to application at field scale (Lin et al. 2022). Soil physico-chemical properties, rain recharge, and geological processes can significantly influence the bioavailability of metal(loid)s in the soil–water matrix and, in turn, affect the phytoremediation performance (Wang et al. 2020a, b). Phytoremediation is a green and nature-based approach with proven success to reduce metal(loid)s mobility in diverse environmental matrixes (Schück and Greger 2020a). Application of phytoremediation using terrestrial plant systems in places, such as contaminated soils affected by mining or metal-processing activities, has been reported in the literature (Khan et al. 2019b; Tisserand et al. 2021; Tognacchini et al. 2020). Similarly, both live and dead macrophytes can be used as biofiltration tools for heavy metals in both natural and constructed wetlands (Bi et al. 2019). Furthermore, they can also be used for final polishing the treated industrial effluents and secondary-treated municipal wastewater (Mustafa and Hayder 2021). The aquatic plants (*C. riparia*, *C. longus*, *C. rotundus*, *I. pseudacorus*, *J. effusus*, *L. salicaria*, *M. aquatica*, *P. australis*, *S. holoschoenus*, and *T. angustifolia*) used in the present study for the remediation of metal(loid)s laden groundwater are also reported to perform phytoremediation of different contaminants, including dyes, pharmaceuticals products, hydrocarbon, poly-halogenated hydrocarbon (Schück and Greger 2020a, b; Khan and Barros 2023). Hence, identifying a proper selection of the macrophytes is a prerequisite for successful tailor-based phytoremediation strategy for contaminated water, soil, and sediments.

Macrophytes can be broadly classified into floating, submerged, and emergent. Macrophytes play an essential role in aquatic ecosystems, as they provide cover for fish and substrate for aquatic invertebrates. Emergent macrophytes are particularly interesting, as they live in amphibious conditions and colonize the margins of water bodies. They are rooted into the substrate but have significant shoot growth above the water level (e.g., *Typha* sp. and *Phragmites* sp.). This facilitates the removal of pollutants from both the water column and the sediment, depending on their compartmental distribution (Mustafa and Hayder 2021). Emergent macrophytes have the capability to accumulate heavy metals from air, water, and sediments using their aerial shoots, submerged leaves, and roots (Nguyen et al. 2021). Macrophytes have demonstrated the ability to grow in salt marshy conditions with tolerance to salt stress and the ability to withstand the effects of high total dissolved solids and multi-metal(loid) contamination (Fernandes et al. 2017). However, their use is partly limited by the lack of commercially available macrophytes with fast growth rates and the ability to tolerate contaminants (Thijs et al. 2016). In addition, the response of macrophytes to contaminants in water and sediments including metal(loids) can vary significantly between species (Schück and Greger 2020b).

The macrophyte application for restoration or remediation purposes must be carefully assessed on a species basis. It was also observed that the provided environmental conditions can also impact the potential of phytoremediation. These factors include, but not limited to, the soil composition, climatic condition, water available, vegetative competition, biological pests (unicellular and multicellular), and availability of light (Wei et al. 2021). Hence, for selection of potent plant, native and readily available plants having higher growth rates and biomass production capacity, presenting a supreme adaptability to the provided environmental condition, should be considered for phytoremediation (Yan et al. 2020). Mohsin et al. (2023) also proposed that native macrophyte *P. australis* and *I. pseudacorus* showed higher potential for phytoremediation of wastewater, contaminated with metal(loid)s, notably accumulating higher levels of Cd in roots than shoots. This is due to potential to grow under provided physical and climatic conditions. Lastly, there is limited to no risk of harming the native ecosystem, as the adopted macrophytes are connatural and are well suited to the given environmental conditions (Leguizamo et al. 2017). Plants display a complex dynamics of metal(loid)s uptake throughout their growth, from early stages to their maturity. Their ability to absorb these elements isn’t static, but rather fluctuates dynamically across various growth phases (Pidlisnyuk et al. 2020). Young plants, with their rapid growth and developing cellular structures, often find themselves more susceptible to the physiological consequences of metal(loid) uptake, as their delicate systems are less equipped to handle the potential disruptions these elements can cause in essential metabolic processes (Angulo-Bejarano et al. 2021). Conversely, mature plants, having already established robust cellular machinery and defence mechanisms, tend to exhibit greater resilience to metal(loid)s exposure (Jamla et al. 2021; Velasco-Arroyo et al. 2024). Furthermore, the duration of exposure itself appears to play a significant role in how plants respond. It is plausible that a short-term encounter with metal(loids) may trigger a distinct set of physiological responses compared to a prolonged exposure (Saleem et al. 2023).

This highlights the complex and multifaceted nature of plant-metal(loid) interactions, where both the developmental stage and the length of exposure influence the plant’s response. In the present work, the main objective was to investigate the tolerance of specific macrophyte species to metal(loid)s in contaminated water, with a focus on how the duration of exposure influences metal uptake, translocation, and the resulting physiological responses, for potential application in phytostabilization or phytorestoration strategies. Plants for contaminated sites should be chosen based on their ability to withstand high pollutant levels, their growth rate, and their ease of acquisition (Schück and Greger 2020a, b). For this purpose, the present study investigates the metal(loid) attenuation capacity of ten native European macrophyte species from central and southern regions. Hence, 10 commercially available native macrophyte species were acquired and exposed to an acidic groundwater containing metal(loid)s at concentrations significantly above the Belgian regulatory levels for groundwater and surface water. The species tested included *C. riparia*, *C. longus*, *C. rotundus*, *I. pseudacorus*, *J. effusus*, *L. salicaria*, *M. aquatica*, *P. australis*, *S. holoschoenus*, and *T. angustifolia.* These selected macrophytes were exposed to these conditions for 5, 10, and 15 days. Plant growth and health were evaluated by means of height, fresh and dry biomass quantification. Tolerance to metal(loid) was additionally investigated by morphology assessment. Finally, determination of metal(loid)s in roots and shoots provided insights into the physiological response of macrophytes to metal(loid)s (uptake vs exclusion) and potential application for phytoremediation strategies (accumulation in roots vs accumulation in shoots).

## Material and methods

### Polluted ground water collection and characterization

Polluted groundwater was collected from an industrial site located in Flanders, Belgium. The range of metal(loid)s in the polluted water extracted from wells located in the industrial site is presented in Supplementary Table 1. The metal(loid) concentrations (in mg L^−1^) of the polluted groundwater (PW) were as follows: Ni 127, Cu 163, Fe 382, Zn 72, As 0.3, Cd 2, and Pb 0.3, respectively, at pH 3.7, and EC 5.3 dS m^−1^. As per the Flemish groundwater sanitation standard the threshold values for metal(loid)s in groundwater (in µg L^−1^) are as follow: Ni 40, Cu 100, Fe 200, Zn 500, As 20, Cd 5, and Pb 20 (Coetsiers et al. 2009). All selected metal(loid)s were above the permissible Flemish groundwater Sanitation Standards. The values of metal(loid)s compared to these sanitation standards was times higher in the following descending order, Ni (3182), Fe (1910), Cu (1632), Cd (441), Zn (143), As (16), and Pb (16).

### Plant material and experimental conditions

Plant seedlings of 10 different species of emergent macrophytes were purchased from Viveros La Dehesa, Valdeobispo, Spain. Plants were maintained in a plant growth room located at the Universidad de Burgos, Spain. To ensure consistent plant characteristics during propagation, strict quality controls were implemented to verify the genetic uniformity of the initial plant stock. This involved purchasing macrophytes primarily derived from vegetative reproduction methods, like rhizomes, turions, or stolons, which inherently maintain the genetic makeup of the parent plant. In the present study, the metal(loid)s attenuation ability of 10 emergent macrophyte species was assessed that are native of the European central and southern regions. A commercially available potting soil was used for the plant cultivation. During the plant's growth, the temperature was maintained at 25:16 °C for day:night, with a photoperiod of 16:8 h light: dark. During the experimentation, uniform-sized plants were used after they had been acclimated for 4 weeks. Each of the selected plants (1-month-old, nearly uniform height) was subjected to treatments (a) polluted groundwater sample (PW) at full strength without any dilution, and (b) controls provided with tap water (CW). The experiment was performed in a continuous batch, using one tray per plant species per treatment. In each tray a total of 9 plant seedling pellets was introduced, among which 3 biological replicates were harvested corresponding to sampling time of respective exposure intervals (5, 10, and 15 days). In each pellet of plant seedings the number of plants were as follows, *C. riparia* 1–2*, C. longus* 2–3*, C. rotundus* 2–3*, I. pseudacorus* 1, *J. effuses* 3–5*, L. salicaria* 3–5, *M. aquatica* 1–2*, P. australis* 1–2, *S. holoschoenus* 1–3*,* and *T. angustifolia* 1*.* Each tray was filled with 2 L of either polluted or tap water, level marked and monitored daily, though out the experiment. Any change in the water level due to evaporation or transpiration was compensated with respective water source. Plants were exposed to these conditions for 5, 10, and 15 days. The duration of experiment with 15 days with interval of 5 days, was enough to find the uptake capacity and comparison of individual species, as many previously published studies were performed to study the tolerance of aquatic plant with similar or even less intervals and duration of time (Schück and Greger 2020a, b; Dean et al. 2022; Newete and Byrne 2016, Newete et al. 2016). At the time of plant harvest, different individual plants (in triplicate) were harvested at each time point (5, 10, and 15 days).

### Harvesting and physiological parameters of plants

Initial and change in plant heights for each exposure interval were noted using images of plants taken at 1, 5, 10, and 15 days, with a 1 × 1 cm square scale and a black background. The image was used for estimation of the plant height using ImageJ. With the help of a wand tool, one by one, measurements of each plant were taken. The combined image for all plants (cultivated on clean and polluted water) is provided in the supplementary information (Supplementary Figs. 1–10). The change in height over the five-day intervals was also calculated to analyse the progression and adaptation of plants as follows:

$$Changes\ in\ height (\%)= \left(\frac{ Height\ in\ specfic\ interval-Initial\ height }{Final\ height}\right)\times 100$$

The fresh and dried biomass of roots and aerial parts for all the plants were quantified gravimetrically and expressed in mg ± mg. For fresh biomass the samples were analysed immediately after harvesting for each respective exposure interval (i.e., 5, 10, and 15 days). The potting soil was removed, and saved for metal(loid)s quantification, while plant roots were rinsed with distilled water to remove any potentially surface linked particles. The rinsed root samples were air dried to remove the excess water adsorbed during washing, and then used for the fresh root biomass quantification. The dried biomass (root and shoot) were noted after dehydration of fresh samples at 60 °C, until constant weight was achieved (~ 96 h). Based on the fresh and dried biomass weight measurements, the water storage capability (g g^−1^) was calculated using the following equation:

$$Water\ storage\ capabilty= \frac{Fresh\ weight-Dried\ weight}{Dried\ weight}$$

### Metal(loid) quantification and plant uptake ability

Water samples were centrifuged, filtered, and acidified using concentrated HNO_3_ (1:9 v:v), whereas dried plant tissues and rhizosphere soil samples were digested. Around 0.25–0.5 g of sample (either soil, root, and aerial part) were weighed and placed in Teflon microwave tubes, to which, 2 mL of H_2_O_2_ (33%) and 8 mL of concentrated nitric acid (65%) were added. Standard (ERM-CD281 Rye grass certified material) and blanks were also included (one for each 50 samples). The comparison for recovery analysis showed high accuracy in measuring metal(loid) concentrations. Among the analysed metal(loid)s, zinc exhibited the highest recovery (98.18%) with a relatively low variability (1.87%), while nickel had the lowest recovery (95.50%) but also a relative variability of 4.78%. Compared to a certified reference material (ERM-CD281 - Rye grass), all measurements had errors within the acceptable range of ± 5% (Supplementary Table 2). Furthermore, the average recovery for all metals was 95.56% with a coefficient of variation (RSD) of only 2.68%. These results demonstrate the accuracy and reproducibility of the analytical method employed. The Teflon tubes were sealed with a Teflon cap and safety disk and samples were digested using an ETHOS ONE microwave digester (Millestone, USA). The digestate was filtered (Scharlau CF/WASH110 filter paper, Ø 110 mm) and diluted with deionized water to make up a final volume of 25 mL. The metal(loid) contents were analysed spectrophotometrically using ICP-OES and ICP-MS. The metal(loid)uptake ability was evaluated by computing the bioaccumulation coefficient (BAC), biological concentration factor (BCF), and translocation factor (TF) as done by Raza et al. (2019), using the following formula:

$$\text{BAC }= {~}^{Metal\;(loid)\;in\;shoot}\!\left/ \!{~}_{Metal\;(loid)\;in\;soil}\right.$$

$$\text{BCF }= {~}^{Metal\;(loid)\;in\;root}\!\left/ \!{~}_{Metal\;(loid)\;in\;soil}\right.$$

$$\text{TF }= {~}^{Metal\;(loid)\;in\;shoot}\!\left/ \!{~}_{Metal\;(loid)\;in\;root}\right.$$

### Statistical analysis

The statistical analysis of data was performed using SPSS v.20. The one-way ANOVA followed by the Duncan’s multiple range test was applied (*p* ≤ 0.05). Mean and standard deviation were calculated using triplicates of the biological data. Stepwise multiple linear regressions (MLRs), after min–max normalization, was performed to assess the relation between the plant dried biomass and the HMs uptake (Khan et al. 2020).

## Results

### Changes in macrophytes’ morpho-physiological status

The morphological examination of the studied macrophytes (Supplementary Figs. 1–10), revealed evident symptoms of decolourisation, chlorosis, necrosis for some plants despite similar biomass and height to controls. This was the case for *C. riparia*, *C. longus*, *C. rotundus*, *I. pseudacorus*, *J. effusus*, and *M. aquatica*, suggesting that these plants did not acclimatize well within the 15 days incubation time. In contrast, the morphology of *P. australis*, *S. holoschoenus*, and *T. angustifolia* was indicative of healthy plants. The details related to the physiological profiles and metal(loid) uptake are discussed in the proceeding sections.

#### Plant height

**Fig. 1:**
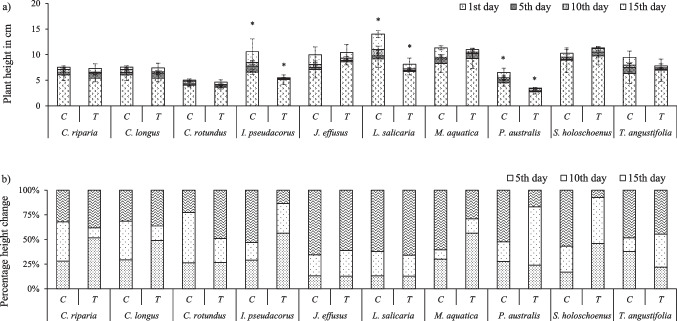
Changes in aquatic plant height exposed to metal(loid) polluted groundwater (T) and in controls (C). a) Total height (cm) of plants at different intervals (0, 5, 10, and 15 days), and b) relative %change in plant height for each time interval

The compiled images taken in successive intervals of 1, 5, 10 and 15 days are presented in the supplementary material (Supplementary Figs. 1–10). These images were used for the estimation of plant height. Figure 1a summarises the total plant height for each of the plants subjected to clean (C) and polluted groundwater (T). After 15 days of exposure total plant heights (cm) were measured. Significant differences were only found for *I. pseudacorus* (5.49 ± 2.49), *L. salicaria* (8.13 ± 3.16)*,* and *P. australis* (3.49 ± 1.27); for these species, plants exposed to contaminated water showed 48, 46, and 46% lower height than their respective controls. For all other species no significant difference were found between treatments (*C. riparia*, *C. longus*, *C. rotundus*, *J. effusus*, *M. aquatica*, *S. holoschoenus*, and *T. angustifolia*)*.* Height and biomass are reliable indicators of a plant’s final growth. In addition, plant performance can be monitored by the growth rate. For this purpose, percentage change in plant heights were also recorded at 5-day intervals (Fig. 1b). On day 10 reduced heights compared to the controls were noted for *C. riparia*, *C. longus, C. rotundus*, *J. effusus*, *L. salicaria*, *M. aquatica* and *T. angustifolia*. However, on day 15 similar heights to the controls were observed suggesting the initial stress due to metal(loid)s and extreme water conditions, while plant recovering if the exposure duration to metal(loid) contamination is increased.

#### Studied plant physical parameters upon metal(loid)s exposure

The studied wetland plants were exposed to polluted groundwater for 15 days and subsequently harvested. Fresh and dried biomass of aerial parts and roots, along with plant height, were quantified (Table 1). Except for *C. riparia* and *M. aquatica,* significant differences in the shoot fresh biomass were observed between the control and the polluted-water treatment. Differences in shoot biomass were positive or negative depending on the plant species. In the case of *C. longus, J. effusus*, and *T. angustifolia* (37,178 ± 3284, 70,992 ± 7788, and 7225 ± 481, respectively in mg) a decrease in fresh shoot biomass of 65, 31, and 35%, as compared to the controls, was recorded. However, for *C. rotundus*, *I. pseudacorus*, *L. salicaria*, *P. australis*, and *S. holoschoenus*, an increase in fresh shoot biomass was observed when plants were exposed to polluted water. The observed fresh biomass (in mg) for these plants was 25,088 ± 3087, 10,6340 ± 2451, 55107 ± 5128, 9940 ± 285, 24195 ± 2591. For plants exposed to contaminated water the increase in fresh shoot biomass was highest for *P. australis* and *S. holoschoenus* (184 and 139%, respectively), while for *C. rotundus*, *I. pseudacorus*, and *L. salicaria* the increment in fresh shoot biomass was 69, 39, and 39%, accordingly. Dried shoot biomass for all the studied plants was found to vary significantly. Interestingly, dried shoot biomass (mg) of *P. australis* (697 ± 72) and *S. holoschoenus* (2072 ± 21) was significantly lower (41 and 13%, respectively) for the water-contaminated treatment in comparison to the controls. The relative increment in dried shoot biomass for plants cultivated with polluted water was in decreasing order: *C. longus* (881%), *I. pseudacorus* (758%), *M. aquatica* (525%), *T. angustifolia* (431%), *C. riparia* (270%), *J. effusus* (87%), *C. rotundus* (37%), and *L. salicaria* (26%). For the water storage capacity (WSC) in the shoot (g g^−1^ DW) all plants showed significant variabilities except *L. salicaria* (5.1 ± 0.9). The plants exposed to contaminated water for the species *C. longus* (0.5 ± 0.1), *T. angustifolia* (1.6 ± 0.1), *M. aquatica* (4.9 ± 0.5), *C. riparia* (1.6 ± 0.3), *I. pseudacorus* (2.6 ± 0.2), and *J. effusus* (3.6 ± 0.7) showed a lower shoot WSC (98, 92, 85, 87, 88, and 68%, respectively) compared to the controls. The opposite trend was observed for the species *C. rotundus* (8.3 ± 1.3), *P. australis* (13.4 ± 2), and *S. holoschoenus* (10.7 ± 1.1). The largest increase in shoot WSC with respect to the controls was observed for *P. australis* and *S. holoschoenus* with an increment of 573 and 231%, respectively. Similarly, an increased shoot WSC of 27% was reported for *C. rotundus* plants exposed to contaminated water, in comparison to the clean water controls.

**Table 1:** Impact on wetland plant biomass and water storage after 15 days with or without exposure to metal(loid)s polluted groundwater

Plant	Exposure^+^	Plants’ root parameters			Plants’ shoot parameters		
		Fresh weight (mg)	Dried weight (mg)	Water storage capacity (g g^−1^ DW)	Fresh weight (mg)	Dried weight (mg)	Water storage capability g g^−1^ DW
*Carex riparia*	C	18,278.67 ± 2816.20^d^	1415.33 ± 186.56^f*^	11.93 ± 1.43^d*^	5337.33 ± 469.45^e*^	1448.67 ± 138.59^ef^	2.69 ± 0.29^b*^
	T	13,403.67 ± 1823.98^ g^	5243.33 ± 206.50^f*^	1.55 ± 0.26^ fg*^	2845.67 ± 565.85^d*^	1269.67 ± 93.04^ cd^	1.27 ± 0.60^b*^
*Cyperus longus*	C	106,435.67 ± 8623.00^a*^	2533.33 ± 57.74^d*^	41.05 ± 3.99^a*^	12,396.33 ± 1327.48^c*^	2533.33 ± 57.74^c^	3.9 ± 0.55^a^
	T	37,178.33 ± 3284.44^e*^	24,842.33 ± 311.20^b*^	0.50 ± 0.12^ g*^	21,994.67 ± 2826.19^b*^	6032 ± 3528.63^b^	3.89 ± 3.50^a^
*Cyperus rotundus*	C	14,860.33 ± 261.25^de*^	1971.33 ± 49.65^def*^	6.54 ± 0.25^e^	7733.67 ± 50.77^d^	1971.33 ± 49.65^d^	2.92 ± 0.11^b^
	T	25,088.33 ± 3086.82^f*^	2699.00 ± 133.59^ g*^	8.32 ± 1.33^c^	8172.0 ± 430.43^c^	2308.33 ± 395.06^ cd^	2.63 ± 0.79^ab^
*Iris pseudacorus*	C	76,258.67 ± 5773.22^b*^	3416.67 ± 733.41^c*^	22.12 ± 5.84^c*^	14,122.33 ± 1804.93^b*^	2750.00 ± 169.00^c*^	4.12 ± 0.36^a*^
	T	106,339.67 ± 2450.73^a*^	29,308.00 ± 1512.96^a*^	2.63 ± 0.15^ef*^	26,602.33 ± 2437.67^a*^	7657.67 ± 462.67^b*^	2.47 ± 0.11^ab*^
*Juncus effusus*	C	103,012.33 ± 3205.48^a*^	8371.00 ± 983.53^a*^	11.39 ± 1.02^d*^	18,595.00 ± 801.62^a*^	8371.00 ± 307.15^a*^	1.22 ± 0.02^c^
	T	70,991.67 ± 7788.44^b*^	15,675.00 ± 1022.59^c*^	3.55 ± 0.71^de*^	28,180.33 ± 1260.10^a*^	11,888.00 ± 1429.86^a*^	1.38 ± 0.17^b^
*Lythrum salicaria*	C	39,731.00 ± 2788.23^d*^	7258.33 ± 533.51^b*^	4.48 ± 0.38^e^	15,269.33 ± 894.93^b*^	7258.33 ± 527.85^b*^	1.11 ± 0.21^c^
	T	55,106.67 ± 5128.11^d*^	9156.33 ± 559.11^e*^	5.06 ± 0.91^d^	10,123.00 ± 981.38^c*^	6106.00 ± 333.88^b*^	0.67 ± 0.25^ab^
*Mentha aquatica*	C	56,582.00 ± 4472.86^c^	1673.33 ± 153.87^ef*^	32.99 ± 4.01^b*^	6110.67 ± 678.83^e*^	1673.33 ± 153.87^de^	2.66 ± 0.41^b^
	T	61,509.00 ± 2827.94^c^	10,457.67 ± 379.70^d*^	4.89 ± 0.48^d*^	4557.00 ± 479.25^d*^	1588.67 ± 218.88^ cd^	1.9 ± 0.44^b^
*Phragmites australis*	C	3498.00 ± 148.01^ g*^	1183.67 ± 142.90^gf*^	1.99 ± 0.45^e*^	3120.33 ± 337.62^f^	1183.67 ± 142.90^f*^	1.64 ± 0.05^c*^
	T	9939.67 ± 284.77^ g*^	697.00 ± 71.58^ g*^	13.40 ± 2.00^a*^	2899.67 ± 347.12^d^	857.67 ± 45.88^d*^	2.37 ± 0.24^ab*^
*Scirpus holoschoenus*	C	10,132.33 ± 763.94^ef*^	2400.00 ± 54.34^de*^	3.22 ± 0.29^e*^	5180.67 ± 468.39^e*^	2400.00 ± 170.16^c*^	1.16 ± 0.07^c*^
	T	24,195.00 ± 2591.00^f*^	2072.33 ± 21.22^ g*^	10.67 ± 1.14^b*^	9437.33 ± 936.91^c*^	3178.33 ± 136.30^c*^	1.98 ± 0.39^ab*^
*Typha angustifolia*	C	11,212.33 ± 97.08^e*^	519.67 ± 48.00^f*^	20.7 ± 1.99^c*^	1985.00 ± 179.35^f^	519.67 ± 48.00^ g*^	2.84 ± 0.44^b^
	T	7224.67 ± 480.89^ g*^	2761.67 ± 97.70^ h*^	1.61 ± 0.09^ fg*^	2264.00 ± 380.14^d^	634.33 ± 40.92^d*^	2.58 ± 0.67^ab^

^+^*C*, control treatment after 15 days, without polluted water exposure, *T*, treatment after 15 days, with polluted water exposure

In super script different alphabets indicate significant difference between different plants, while “*” indicate significant difference in response between control and treatments within the same plant. Data is presented in Mean ± SD, with *n* = 3, at *p* < 0.05

Significant differences were also observed for the roots, between controls and plants exposed to contaminated water. For fresh root biomass (mg) significant differences were observed between treatments for all species except for *C. rotundus* (8172 ± 430) and *P. australis* (2890 ± 347). A reduction in the fresh root biomass of *C. riparia* (2846 ± 566), *L. salicaria* (10,123 ± 981), and *M. aquatica* (4557 ± 479) was observed for the contaminant-exposed treatments compared to the controls. Here, plants exposed to contaminated water produced 47, 34, and 25% less biomass, respectively, compared to plants grown with clean water. Fresh root biomass was significantly larger for *I. pseudacorus* (26,602 ± 2438), *S. holoschoenus* (9437 ± 937), *C. longus* (21,995 ± 2826), *J. effusus* (28,180 ± 1260), and *T. angustifolia* (2264 ± 380). For these plants a relative increase of 88, 82, 77, 51, and 14% was recorded compared to the controls. The root-dried weights of *C. riparia*, *C. longus*, and *C. rotundus* did not differ between treatments. In contrast, for *I. pseudacorus* (7658 ± 463), *J. effusus* (11,888 ± 1430)*, S. holoschoenus* (3178 ± 136), and *T. angustifolia* (634 ± 41) a relative increase of 78, 42, 32, and 22% higher dried root biomass (mg) was recorded for plants exposed to metal(loid)s with respect to the controls. For *C. longus*, *C. rotundus*, *I. pseudacorus*, *J. effusus*, *L. salicaria*, and *M. aquatica*, no significant changes were noted for the root WSC between plants exposed to metal(loid)s and the controls. For *C. riparia*, *I. pseudacorus*, and *T. angustifolia* the root WSC decreased by 53, 40, and 9% in plants exposed to contaminated water compared to the controls. The opposite trend was observed for *S. holoschoenus* and *P. australis* with an increase in root WSC of 71 and 45%, respectively, in plants exposed to contaminated water.

Higher cumulative fresh root biomass was observed on day 15 for *S. holoschoenus* (120%), *P. australis* (94%), *C. rotundus* (47%), *I. pseudacorus* (47%), *L. salicaria* (19%), and *M. aquatica* (5.4%) plants exposed to contaminated water than for the controls. The opposite was reported for *C. longus* (− 18%), *C. riparia* (− 28%), *T. angustifolia* (− 31%), and *J. effusus* (− 50%). Higher cumulative dried root biomass was observed on day 15 for *C. longus* (509%), *I. pseudacorus* (499%), *M. aquatica* (260%), *T. angustifolia* (227%), *C. riparia* (127%), *J. effusus* (65%), *C. rotundus* (27%), *S. holoschoenus* (9.4%), and *L. salicaria* (5.1%) plants exposed to contaminated water than for the controls. *P. australis* was the only species with lower cumulative root dried biomass in plants exposed to contaminated groundwater (− 34%) compared to the control. The water storage capability (%) on day 15 was higher for *P. australis* (335), *S. holoschoenus* (189), *C. rotundus* (16), and *L salicaria* (2.258) and lower for *C. longus* (90), *T. angustifolia* (− 82)*, M. aquatica* (− 81), *C. riparia* (− 81)*, I. pseudacorus* (− 81), and *J. effusus* (− 60.82) in plants exposed to contaminated water than in the controls.

### Meta(loid)s concentration in macrophytes, rhizospheric soil and groundwater

**Fig. 2:**
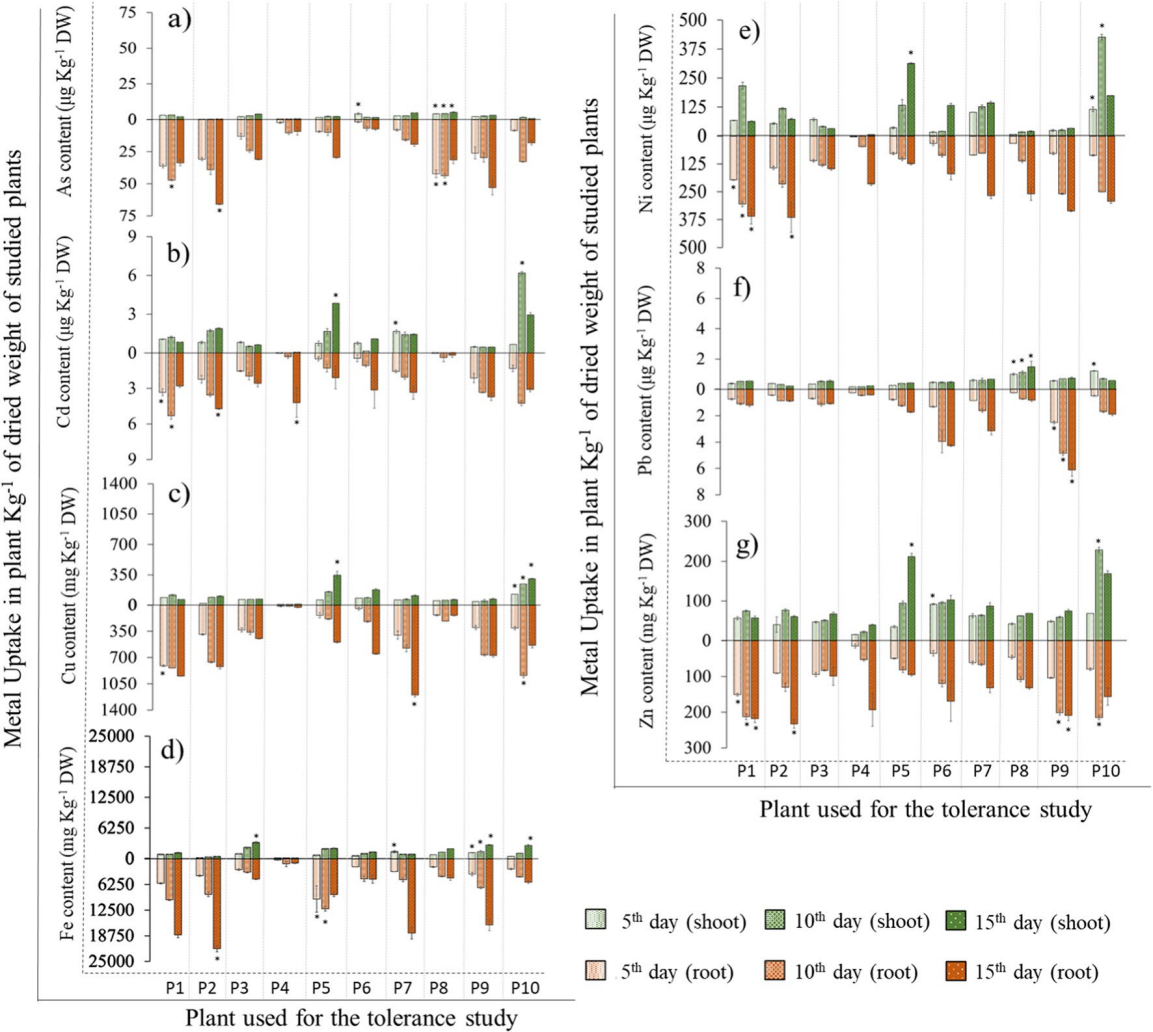
Metal(loid)s uptake profile for the shoots (green bars) and roots (brown bar) of the exposed aquatic plants to polluted groundwater. The x-axis represents the different plants from left to right P1) *C. riparia*, P2) *C. longus*, P3) *C. rotundus*, P4) *I. pseudacorus*, P5) *J. effusus*, P6) *L. salicaria*, P7) *M. aquatica*, P8) *P. australis*, P9) *S. holoschoenus*, and P10) *T. angustifolia.* The y-axis represents the increase in metal(loid) concentrations for the shoots (green bar upwards) and for the roots (brown bar downwards). a) As-, b) Cd-, c) Cu-, d) Fe-, e) Ni-, f) Pb-, and g) Zn-content (in mg or µg per Kg of plant dried weight). Each bar is the mean of 3 values ± SD. Asterisks (*) on bars indicate a significant difference for the highest metal(loid) uptake for each corresponding element on the specified day (5, 10, or 15^th^) at *p* < 0.05

The results of metal(loid)s uptake and compartmentalization are presented in Fig. 2. Additional details related to the statistical analysis are provided in Supplementary Table 3 (for the shoot) and 4 (for the root). Among the studied plants, one of the most prominent features observed was that the largest quantity of metal(loid)s generally was stabilized in the roots rather than in the shoots of the plants. However, arsenic (As), in μg kg^−1^ of the plant’s dried weight (DW), was found to be significantly highest in the aerial parts of *P. australis* (4.0 ± 0.1, 4.1 ± 0.2, and 5.1 ± 0.5, on the 5th, 10th, and 15th day of exposure, respectively). Similarly, *P. australis* also showed the highest As uptake in the root on the 5th and 10th day with 42.3 ± 2.9 and 43.7 ± 1.7 μg kg^−1^ DW, respectively. Alongside with *P. australis*, *C. riparia* also showed higher As root uptake on the 10th day (47.1 ± 0.4), while only *C. longus* showed higher As uptake in root on the 15th day (65.9 ± 0.3) (Fig. 2a). The Cd uptake in the shoots and the roots showed a variable pattern depending on the species with highest levels for *J. effusus* on the 15th day (3.8) compared to other plants. For the roots, the highest level of Cd (μg kg^−1^ DW) was reported for *C. longus*, *I. pseudacorus* and *P. australis* on the 15th day (ranging between 4 and 5.5) (Fig. 2b). For Cu uptake (mg kg^−1^ DW) in the shoots, *T. angustifolia* showed the highest concentrations of all species at each successive exposure interval, 125.4 ± 0.9, 244.4 ± 0.4, and 303.1 ± 5.4, respectively. *J. effusus* also showed a significantly higher shoot uptake on the 15th day (347.1 ± 44.6 mg kg^−1^ DW). For the roots no successive Cu uptake trend was observed over time; the highest concentration (1199) was reported on the 15th day for *M. aquatica* (Fig. 2c). *S. holoschoenus* showed significantly higher Fe (mg kg^−1^ DW) uptake in the shoot at each interval compared to the other plants, with highest Fe concentration (2842 ± 113) on the 15th day. For root Fe uptake the highest values (mg kg^−1^ DW) were noted for *J. effusus* with 9758 ± 3224 on the 5th day and 12,132 ± 618 on the 10th day as well as for *C. longus* (21,859 ± 784) on the 15th day (Fig. 2d). The highest Ni uptake in the shoots was noted for *J. effusus* (314 ± 2) on the 15th day. For the roots the highest Ni uptake was recorded generally for *C. riparia* and *C. longus* at each time interval with a maximum value of ~ 360 on the 15th day (Fig. 2e). The highest Pb uptake (μg kg^−1^ DW) in the shoot was noted for *P. australis* with a maximum concentration of 1.5 ± 0.4 on day 15. For Pb uptake in roots, *S. holoschoenus* showed a significantly higher increase at all time intervals compared to other species, with a maximum value of 6.1 ± 0.5 (μg kg^−1^ DW) on 15th day (Fig. 2f). The Zn uptake in the shoot (mg kg^−1^ DW) was variable among the different plants (Fig. 2g). The highest Zn uptake was recorded for *J. effusus* on the 15th day (212 ± 8). For the roots the highest Zn uptake was observed for *C. riparia* with 218 ± 11, on day 15. In addition, high Zn root uptake in mg kg^−1^ DW was reported for *C. longus* (233 ± 12) on day 15, for *S. holoschoenus* (201 ± 7, and 209 ± 15, respectively) on days 10 and 15, as well as for *T. angustifolia* (215 ± 6) on day 10.

The profiles for metal(loid) content in the rhizosphere of the macrophytes is presented in Table 2. Among the most notable trend was the successive increase in the metal(loid) concentration in the rhizospheric soil as time progressed, with the highest stabilization on day 15. An exception to this was As; here, the highest stabilization was achieved by *I. pseudacorus* on day 5. The highest As rhizospheric retention (77 ± 4 μg kg^−1^ DW) was performed by *J. effusus*. For Cd stabilization in rhizospheric soil (μg kg^−1^ DW), the best performing plants were *L. salicaria*, *C. longus*, *M. aquatica*, and *P. australis* revealed significantly with 24 ± 4, 26 ± 1, 24 ± 1, and 23 ± 2, respectively. *J. effusus* and *L. salicaria* showed the highest rhizospheric stabilization for Cu (2529 ± 215, and 2694 ± 137 mg kg^−1^ DW, respectively). The highest stabilization for Fe in soil was observed for *J. effusus* with 9826 ± 74 mg kg^−1^ DW. *L. salicaria* significantly showed the highest concentrations in rhizospheric soil for both Ni (1835 ± 146 μg kg^−1^ DW) and Zn (798 ± 85 mg kg^−1^ DW). For Pb, the highest soil stabilization was noted for *I. pseudacorus* (9 μg kg^−1^ DW).

**Table 2:** Metal(loid) concentrations in the rhizospheric soil of the studied aquatic plants grown in polluted groundwater

Plant	Days^+^	As^1^	Cd^1^	Cu^2^	Fe^2^	Ni^1^	Pb^1^	Zn^2^
*Carex riparia*	5	1.97 ± 0.17^c^	0.05 ± 0.01^c^	11.45 ± 0.71^c^	2026.17 ± 83.76^b^	1.43 ± 0.01^c^	1.62 ± 0.14^a^	15.65 ± 2.14^c^
	10	2.98 ± 0.11^b^	4.75 ± 0.11^b^	321.39 ± 11.90^b^	2734.20 ± 330.02^b^	339.73 ± 3.92^b^	1.74 ± 0.03^a^	157.61 ± 3.16^b^
	15	38.96 ± 0.69^a^	11.20 ± 1.25^a^	690.16 ± 11.08^a^	4862.59 ± 283.30^a^	656.93 ± 7.51^a^	1.85 ± 0.03^a^	333.25 ± 7.78^a^
*Cyperus longus*	5	2.13 ± 0.39^b^	7.19 ± 1.35^b^	1051.22 ± 111.50^a^	1928.70 ± 51.64^b^	587.73 ± 24.78^b^	1.34 ± 0.27^a^	212.30 ± 29.17^b^
	10	6.24 ± 0.13^b^	20.52 ± 1.46^a^	1135.59 ± 132.16^a^	4017.26 ± 39.66^a^	1125.99 ± 37.57^a^	1.58 ± 0.29^a^	524.75 ± 16.48^a^
	15	44.28 ± 1.38^a^	23.45 ± 3.71^a*^	1132.99 ± 63.73^a^	5615.92 ± 300.62^a^	1297.67 ± 7.59^a^	1.81 ± 0.20^a^	616.49 ± 39.34^a^
*Cyperus rotundus*	5	4.52 ± 1.28^b^	5.33 ± 0.58^b^	268.43 ± 107.81^c^	2653.39 ± 366.03^b^	308.51 ± 21.83^b^	1.37 ± 0.03^a^	176.89 ± 0.69^b^
	10	5.40 ± 0.69^b^	7.49 ± 1.49^ab^	700.04 ± 164.26^a^	4033.69 ± 154.02^a^	459.32 ± 79.22^b^	1.30 ± 0.20^a^	278.54 ± 111.14^a^
	15	31.03 ± 5.97^a^	11.56 ± 4.24^a^	421.20 ± 179.63^b^	4956.11 ± 1277.00^a^	767.23 ± 77.41^a^	1.54 ± 0.27^a^	376.71 ± 21.27^a^
*Iris pseudacorus*	5	24.22 ± 0.29^a^	0.16 ± 0.02^c^	12.17 ± 1.92^b^	5199.47 ± 117.33^b^	11.77 ± 0.88^b^	8.83 ± 0.65^a^	37.15 ± 1.83^b^
	10	9.60 ± 0.57^b^	2.12 ± 0.00^b^	16.45 ± 2.31^b^	3449.06 ± 245.80^a^	58.71 ± 5.72^b^	8.72 ± 0.49^a^	44.88 ± 2.42^b^
	15	10.52 ± 0.68^b^	11.27 ± 1.56^a^	79.19 ± 1.63^a^	3659.39 ± 27.56^a^	731.62 ± 148.93^a^	9.32 ± 0.07^a*^	284.74 ± 5.12^a^
*Juncus effusus*	5	10.66 ± 2.00^c^	15.25 ± 2.70^b^	1344.24 ± 21.24^b^	4472.29 ± 424.02^b^	849.55 ± 85.42^a^	2.64 ± 0.12^a^	401.36 ± 4.49^b*^
	10	27.06 ± 2.32^b^	16.34 ± 1.91^b^	1501.00 ± 168.33^b^	4643.85 ± 596.38^b^	945.80 ± 68.06^a^	2.79 ± 0.17^a^	431.03 ± 34.53^ab^
	15	76.68 ± 3.92^a*^	20.09 ± 2.13^a^	2528.64 ± 214.63^a*^	9826.24 ± 73.96^a*^	1178.52 ± 31.13^a^	2.66 ± 0.12^a^	518.61 ± 29.35^a^
*Lythrum salicaria*	5	12.59 ± 1.48^b^	12.36 ± 0.54^b^	656.63 ± 0.06^b^	5634.94 ± 140.33^b^	413.59 ± 9.80^b^	6.05 ± 0.37^a^	232.21 ± 11.92^b^
	10	15.00 ± 0.31^ab^	20.23 ± 2.03^a^	2402.00 ± 113.89^a^	7097.61 ± 165.48^a^	1674.54 ± 100.76^a^	6.53 ± 0.40^a^	576.63 ± 18.16^a^
	15	19.85 ± 0.91^a^	26.52 ± 1.23^a*^	2694.17 ± 137.05^a*^	8119.91 ± 335.45^a^	1834.80 ± 145.73^a*^	6.46 ± 0.29^a^	798.32 ± 84.77^a*^
*Mentha aquatica*	5	2.51 ± 0.26^b^	3.54 ± 0.46^c^	141.17 ± 6.53^c^	2186.13 ± 241.56^b^	202.91 ± 10.93^c^	1.53 ± 0.08^a^	112.70 ± 9.67^c^
	10	3.48 ± 0.17^b^	10.03 ± 0.50^b^	677.08 ± 64.99^b^	2492.30 ± 4.79^b^	536.32 ± 24.92^b^	1.58 ± 0.20^a^	342.75 ± 10.54^b^
	15	20.71 ± 0.57^a^	24.14 ± 0.90^a*^	1563.38 ± 135.28^a^	3675.09 ± 155.97^a^	1423.67 ± 90.33^a^	2.08 ± 0.00^a^	697.17 ± 8.10^a^
*Phragmites australis*	5	3.08 ± 0.03^b^	2.90 ± 0.01^c^	480.68 ± 28.10^b^	3741.92 ± 290.72^b^	185.08 ± 7.15^c^	1.12 ± 0.09^b^	180.20 ± 9.82^c^
	10	3.90 ± 0.66^b^	9.75 ± 1.20^b^	585.13 ± 80.54^b^	4456.58 ± 319.15^ab^	440.92 ± 47.57^b^	1.30 ± 0.18^b^	250.18 ± 14.38^b^
	15	20.02 ± 3.72^a^	23.28 ± 1.79^a*^	1244.71 ± 24.15^a^	4741.13 ± 238.13^a^	1255.47 ± 100.68^a^	2.00 ± 0.27^a^	547.70 ± 30.30^a^
*Scirpus holoschoenus*	5	6.94 ± 0.05^b^	5.89 ± 0.76^b^	443.93 ± 0.10^b^	4572.71 ± 150.38^b^	284.89 ± 9.63^c^	1.16 ± 0.10^a^	287.33 ± 5.60^c^
	10	7.27 ± 0.55^b^	9.65 ± 1.25^b^	1108.76 ± 106.46^a^	4934.39 ± 231.92^ab^	596.92 ± 50.66^b^	1.78 ± 0.08^a^	422.23 ± 20.64^b^
	15	36.52 ± 1.52^a^	12.17 ± 0.38^a^	1350.89 ± 35.49^a^	5212.13 ± 126.02^a^	708.36 ± 58.15^a^	1.90 ± 0.10^a^	527.03 ± 15.57^a^
*Typha angustifolia*	5	1.49 ± 0.19^b^	2.43 ± 0.28^c^	178.52 ± 7.67^c^	7093.48 ± 57.74^b^	179.46 ± 3.03^c^	1.04 ± 0.04^a^	136.00 ± 13.96^c^
	10	2.42 ± 0.31^b^	5.14 ± 0.16^b^	273.83 ± 20.39^b^	7470.67 ± 292.62^ab^	292.89 ± 11.50^b^	1.29 ± 0.06^a^	218.13 ± 16.53^b^
	15	39.93 ± 0.99^a^	10.82 ± 1.41^a^	637.41 ± 44.27^a^	8148.07 ± 713.84^a^	659.64 ± 14.75^a^	1.51 ± 0.14^a^	346.00 ± 1.84^a^

^+^Days of Exposure. In super script alphabets indicate significant difference between different exposure duration (5, 10, or 15 days) of the specified aquatic plant’s soil with “a” being highest followed by later alphabets, while “*” indicate significant highest metal(loid)s’ uptake among different aquatic plant within all duration of exposure (5, 10, and 15 days)

^1^The values of As, Cd, Ni, and Pb were presented in μg kg^−1^ of the plant dried biomass

^2^For Cu, Fe, and Zn the values were presented in mg kg^−1^ of the plant dried biomass

**Fig. 3:**
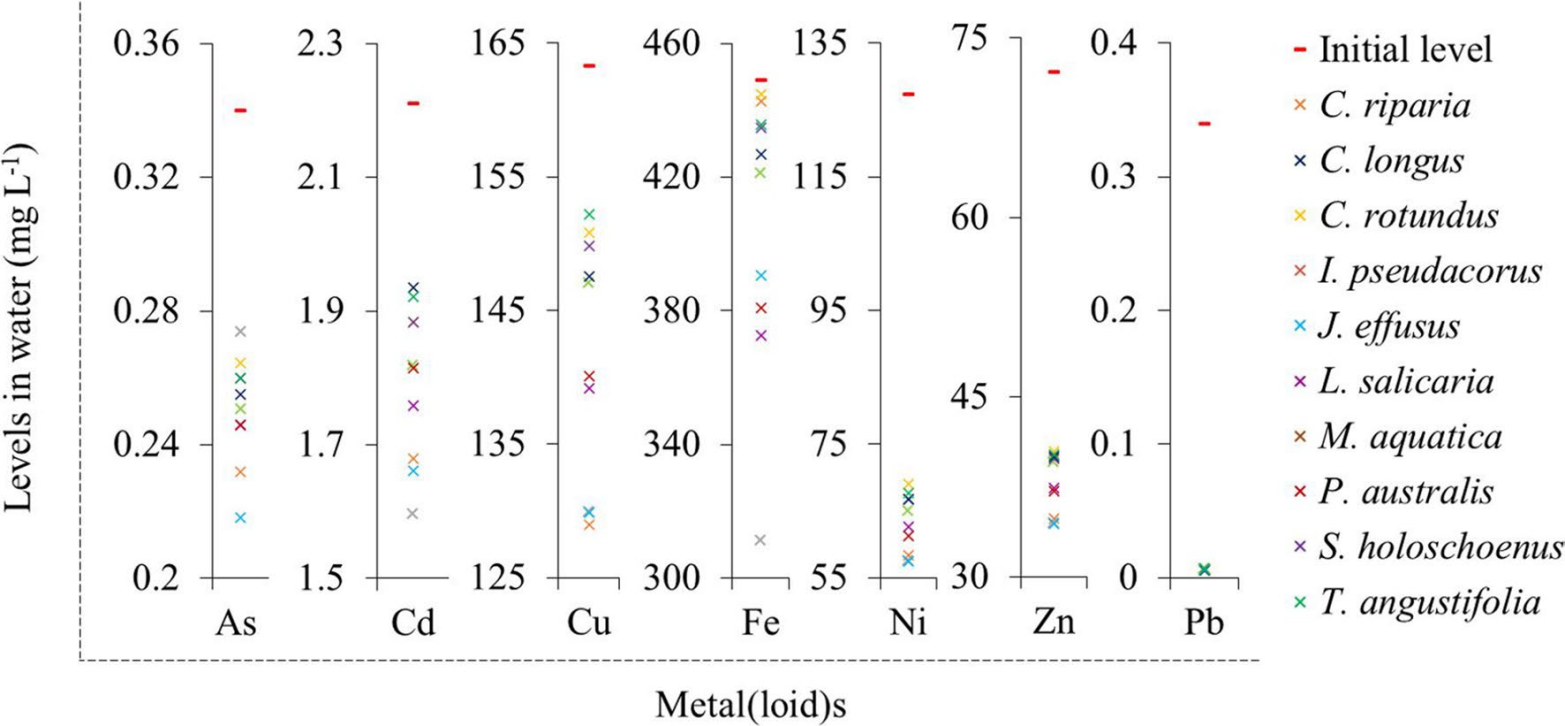
Concentrations of metal(loid)s in groundwater on the 15th day of exposure for the respective plant treatment. The x-axis represents the different metal(loid)s; the y-axis the respective metal(loid) concentrations in mg L^−1^. The red line indicates the initial concentration at the start of the experiment (0 day), while the different coloured cross signs indicate the residual metal(loid) concentration in the groundwater for the respective plants

The concentrations of metal(loid)s remaining in the polluted groundwater after the 15 days exposure time are presented in Fig. 3. A significant reduction in metal(loid) concentrations in water was observed for most species studied because of either translocation into the plants, precipitation/adsorption in the rhizospheric soil or a combination of the two. The As was found lowest (~ 0.22 mg L^−1^) in ground water with *J. effusus,* however, this retention was mainly in the rhizospheric soil (Table 2). For Cd, the lowest level (1.60 ± 0.03 mg L^−1^) in treated groundwater were noted for the *I. pseudacorus* and seeing the rhizospheric level, the Cd is translated in the plant (Fig. 2, Table 4). The Cu levels were lowest (~ 130 mg L^−1^) with *I. pseudacorus*, *J. effusus,* and *C. riparia*. The primary reason for this removal was due to plant uptake for *C. riparia*, while for *I. pseudacorus*, and *J. effusus*. Ni levels were lowest (~ 57 mg L^−1^) and were found for *C. riparia*, *I. pseudacorus,* and *J. effusus* which due to both soil adsorption and plant uptake. For Zn the lowest levels were noted (~ 33 mg L^−1^) with for *C. riparia*, *I. pseudacorus*, and *J. effusus*, that was mainly due to plant uptake of Zn in the roots. The ranges of percentage of metal(loid) removal from the contaminated groundwater after cultivation of wetland plants for 15 days was as follows: As (19–36%), Cd (12–28%), Cu (7–21%), Fe (1–31%), Ni (46–55%), Pb (97–98%), and Zn (44–52%).

### MLR analysis on the impact of metal(loid) uptake and polluted water exposure duration in the shoot and root compartments

The impact of specific metals on plant biomass was assessed by means of multiple linear regression (MLR) equations considering metal(loid) uptake and duration of exposure as independent variables and dried plant biomass as dependent variable. Results are displayed in Supplementary Fig. 11 for shoots and Supplementary Fig. 12 for roots. In this model, a *p* < 0.05 was considered statistically significant, and a coefficient of regression (*R*^2^) with a value close to 1 was considered a good/strong model fit. The data used for this analysis were taken from plant dried biomass (compartment-wise) at the interval of 5, 10, and 15 days of exposure. The number of observations (*n*) for dependent and independent variables was 9. The Supplementary Fig. 11a–j displays the MLR models for shoots. For shoot biomass the least favourable model fit *R*^2^ were 0.659 for *I. pseudacorus*, 0.752 for *S. holoschoerus* and 0.811 for *L. salicaria.* For all other species the model fit *R*^2^ ranged between 0.892 for *C. rotundus* and 0.990 for *J. effesus*. The contribution of metal concentrations and exposure time to the total model variability differed significantly between species. On occasion, one single variable accounted for more than 50–75% of the total data variability. This was the case for *C. riparia* (Cu), *C. longus* (Fe), *I. pseudacorus* (Fe) and *L. salicaria* (exposure time). For the other species at least two but generally three or more variables accounted for > 75% of the model variability. Supplementary Fig. 12a–j displays the MLR models for roots. Here the model fit *R*^2^ was > 0.96 for all species. As for the shoots the contribution of metal concentrations and exposure time to the total model variability differed significantly between species. In addition, no common pattern was observed for the roots and shoots of the same species, that is, the variables accounting for most of the variability observed in the shoots were not necessarily the same ones those reported for the roots. For *C. riparia* and *C. rotundus* Fe accounted for approximately 50% of the total variability observed, whereas for *I. pseudacorus* the same was observed for As. For the other species at least two but generally three or more variables accounted for > 50% of the model variability.

### Bioaccumulation factor, bioconcentration factor and translocation factor of the studied macrophytes

The bioaccumulation factor (BAF) for each metal(loid) and plant on days 5, 10, and 15 are shown in Table 3. For As, the highest BAF was noted generally on the 5th or 10th day of exposure, except in the case of *I. pseudacorus.* The highest BAF for As (1.3–1.5) on the 5th and on the 10th day (1.1) was recorded for *C. riparia* and *P. australis*; on the 15th day for *M. aquatica* (0.2). The highest BAF for Cd on the 10th (1.2) and 15th day (0.3) was observed for *T. angustifolia*. Similarly, *T. angustifolia* also showed the highest BAF for Cu on days 10 (0.9) and 15 days (0.5) as well as for Ni with 1,5 on day 10 and 0.3 on day 15. Likewise, *J. effusus* exhibited a BAF of 0.3 for Ni on day 15. *C. rotundus* (0.5) and *S. holoschoenus* (0.7) exhibited the highest BAF for Fe on day 15. For Pb, the highest BAF on day 5 was noted for *T. angustifolia* (1.2), while *P. australis* showed the highest BAF values on the 10th and 15th days (0.9 and 0.7, respectively).

**Table 3:** Bioaccumulation factors (BAF) of the studied wetland plants after 5, 10, and 15 days exposure to metal(loid)s polluted groundwater

Plant	Days^+^	As	Cd	Cu	Fe	Ni	Pb	Zn
*Carex riparia*	5	1.516 ± 0.170^a*^	24.049 ± 7.714^a*^	7.522 ± 0.194^a*^	0.412 ± 0.040^a^	47.069 ± 1.210^a*^	0.233 ± 0.030^a^	3.680 ± 0.784a
	10	1.066 ± 0.012^a*^	0.256 ± 0.021^b^	0.362 ± 0.038^b^	0.317 ± 0.038^ab^	0.639 ± 0.052^b^	0.299 ± 0.009^a^	0.475 ± 0.005b
	15	0.050 ± 0.000^b^	0.075 ± 0.007^c^	0.095 ± 0.004^c^	0.235 ± 0.036^b^	0.095 ± 0.002^c^	0.290 ± 0.008^a^	0.171 ± 0.016^c^
*Cyperus longus*	5	0.108 ± 0.013^a^	0.117 ± 0.035^a^	0.020 ± 0.001^b^	0.064 ± 0.002^a^	0.091 ± 0.010^a^	0.295 ± 0.076^a^	0.184 ± 0.063^a^
	10	0.041 ± 0.004^b^	0.085 ± 0.007^b^	0.080 ± 0.007^a^	0.068 ± 0.002^a^	0.105 ± 0.007^a^	0.198 ± 0.042^ab^	0.146 ± 0.002^a^
	15	0.009 ± 0.000^c^	0.082 ± 0.014^b^	0.091 ± 0.010^a^	0.071 ± 0.002^a^	0.056 ± 0.003^b^	0.111 ± 0.002^b^	0.098 ± 0.002^b^
*Cyperus rotundus*	5	0.445 ± 0.121^a^	0.157 ± 0.027^a^	0.276 ± 0.121^a^	0.359 ± 0.062^a^	0.230 ± 0.019^a^	0.244 ± 0.011^b^	0.265 ± 0.014^a^
	10	0.479 ± 0.082^a^	0.068 ± 0.009^b^	0.099 ± 0.026^b^	0.564 ± 0.028^b*^	0.089 ± 0.009^b^	0.405 ± 0.077^a^	0.203 ± 0.073^a^
	15	0.122 ± 0.017^b*^	0.058 ± 0.020^b^	0.191 ± 0.096^ab^	0.689 ± 0.139^b*^	0.041 ± 0.003^b^	0.360 ± 0.102^a^	0.179 ± 0.017^a^
*Iris pseudacorus*	5	0.014 ± 0.000^b^	0.113 ± 0.017^a^	0.751 ± 0.148^a^	0.002 ± 0.000^b^	0.008 ± 0.001^a^	0.018 ± 0.001^a^	0.417 ± 0.024^a^
	10	0.036 ± 0.005^a^	0.010 ± 0.000^b^	0.561 ± 0.116^a^	0.009 ± 0.001^a^	0.002 ± 0.000^b^	0.020 ± 0.001^a^	0.494 ± 0.026^a^
	15	0.050 ± 0.004^a^	0.005 ± 0.000^b^	0.155 ± 0.015^b^	0.011 ± 0.000^a^	0.009 ± 0.003^a^	0.023 ± 0.001^a^	0.139 ± 0.007^b^
*Juncus effusus*	5	0.138 ± 0.023^a^	0.049 ± 0.013^b^	0.043 ± 0.004^b^	0.150 ± 0.014^b^	0.042 ± 0.008^b^	0.093 ± 0.006^b^	0.087 ± 0.007^c^
	10	0.077 ± 0.019^b^	0.103 ± 0.013^a^	0.103 ± 0.010^ab^	0.433 ± 0.072^a^	0.141 ± 0.020^ab^	0.137 ± 0.012^a^	0.220 ± 0.017^b^
	15	0.027 ± 0.004^c^	0.193 ± 0.023^a^	0.137 ± 0.009^a^	0.212 ± 0.008^b^	0.266 ± 0.008^a*^	0.155 ± 0.010^a^	0.410 ± 0.035^a^
*Lythrum salicaria*	5	0.324 ± 0.092^a^	0.061 ± 0.008^a^	0.125 ± 0.005^a^	0.098 ± 0.002^b^	0.040 ± 0.006^b^	0.074 ± 0.007^a^	0.398 ± 0.027^a^
	10	0.108 ± 0.014^ab^	0.007 ± 0.000^b^	0.036 ± 0.006^b^	0.139 ± 0.008^a^	0.012 ± 0.001^c^	0.069 ± 0.010^a^	0.167 ± 0.008^b^
	15	0.073 ± 0.007^b^	0.041 ± 0.002^a^	0.065 ± 0.009^b^	0.158 ± 0.004^a^	0.072 ± 0.003^a^	0.072 ± 0.003^a^	0.131 ± 0.024^b^
*Mentha aquatica*	5	1.009 ± 0.109^a^	0.475 ± 0.083^a^	0.419 ± 0.046^a^	0.641 ± 0.136^a*^	0.506 ± 0.026^a^	0.380 ± 0.044^a^	0.561 ± 0.070^a^
	10	0.766 ± 0.039^ab^	0.141 ± 0.014^b^	0.100 ± 0.020^b^	0.346 ± 0.015^b^	0.236 ± 0.004^b^	0.377 ± 0.136^a^	0.186 ± 0.010^b^
	15	0.225 ± 0.006^b*^	0.059 ± 0.004^c^	0.070 ± 0.006^b^	0.228 ± 0.008^b^	0.101 ± 0.005^c^	0.312 ± 0.010^a^	0.125 ± 0.012^b^
*Phragmites australis*	5	1.281 ± 0.052^a*^	0.004 ± 0.000^a^	0.103 ± 0.003^a^	0.223 ± 0.011^b^	0.040 ± 0.002^a^	0.881 ± 0.092^a^	0.236 ± 0.020^a^
	10	1.088 ± 0.214^a*^	0.002 ± 0.000^a^	0.096 ± 0.012^ab^	0.304 ± 0.013^ab^	0.038 ± 0.006^a^	0.870 ± 0.197^a*^	0.250 ± 0.017^a^
	15	0.255 ± 0.023^b^	0.001 ± 0.000^a^	0.050 ± 0.004^b^	0.437 ± 0.022^a^	0.016 ± 0.001^b^	0.732 ± 0.084^a*^	0.126 ± 0.006^b^
*Scirpus holoschoenus*	5	0.297 ± 0.011^a^	0.081 ± 0.015^a^	0.093 ± 0.003^a^	0.272 ± 0.014^b^	0.084 ± 0.017^a^	0.474 ± 0.011^a^	0.169 ± 0.006^a^
	10	0.323 ± 0.020^a^	0.048 ± 0.008^b^	0.043 ± 0.014^b^	0.293 ± 0.019^b^	0.043 ± 0.008^b^	0.384 ± 0.015^a^	0.139 ± 0.008^a^
	15	0.079 ± 0.005^b^	0.037 ± 0.003^b^	0.051 ± 0.007^b^	0.545 ± 0.019^a*^	0.047 ± 0.003^b^	0.381 ± 0.047^a^	0.142 ± 0.009^a^
*Typha angustifolia*	5	0.150 ± 0.020^ab^	0.276 ± 0.035^b^	0.703 ± 0.036^a^	0.070 ± 0.007^b^	0.646 ± 0.064^ab^	1.158 ± 0.033^a*^	0.506 ± 0.051^b^
	10	0.595 ± 0.066^a^	1.205 ± 0.062^a*^	0.896 ± 0.070^a*^	0.152 ± 0.009^ab^	1.456 ± 0.034^a*^	0.538 ± 0.041^b^	1.053 ± 0.051^a^
	15	0.016 ± 0.000^b^	0.275 ± 0.040^a^	0.477 ± 0.030^b*^	0.341 ± 0.046^a^	0.264 ± 0.005^b*^	0.379 ± 0.032^b^	0.488 ± 0.024^b^

^+^Days of Exposure. In super script alphabets indicate significant difference between bioaccumulation factor of the different studied plants with “a” being highest followed by later alphabets. Data is presented in Mean ± SD, with n = 3, at *p* < 0.05. Asterisk (*) on bars indicates significantly highest metal(loid) uptake for each corresponding metal on specified day (5, 10, or 15th)

The bioconcentration factors (BCF) for each metal(loid) and plant on days 5, 10, and 15 are shown in Table 4. For As, the BCF increased as time progressed, except in the case of *I. pseudacorus* and *L. salicaria.* The highest BCF for As on day 15 was found for *C. longus*, *P. australis*, and *S. holoschoenus* (~ 1.5). For Cd, the highest BCFs (~ 0.4) on day 15 was recorded for *I. pseudacorus*. For Cu the BCF increase in time was observed only for *C. longus*, *J. effusus*, and *L. salicaria*. The greatest BCF for Cu on day 15 was noted for *C. riparia* (1.4) and *C. rotundus* (1.2). For Fe a BCF increase in time was observed for all plants except for *J. effusus*. *M. aquatica* showed the highest BCF for Fe (4.9) on day 15. The highest BCF for Pb was found on days 10 and 15 for all plants. The highest BCF for Fe was recorded for *S. holoschoenus* (2.2–3.2). Interestingly, the highest BCF for Ni (137) and Zn (9.75) were observed for *C. riparia* on day 5, whereas the BCF significantly decrease to ≤ 1 on days 10 and 15.

**Table 4:** Bioconcentration factor (BCF) of the studied wetland plants after 5, 10, and 15 days exposure to metal(loid)s polluted groundwater

Plant	Days^+^	As	Cd	Cu	Fe	Ni	Pb	Zn
*Carex riparia*	5	18.421 ± 1.042^a*^	75.432 ± 22.170^a*^	70.796 ± 3.534^a*^	2.922 ± 0.087^a*^	137.116 ± 2.031^a*^	0.460 ± 0.077^b^	9.750 ± 1.349^a*^
	10	15.834 ± 0.721^a*^	1.118 ± 0.052^b*^	2.611 ± 0.103^b^	3.692 ± 0.486^a*^	0.898 ± 0.024^b*^	0.635 ± 0.033^a^	1.350 ± 0.049^b*^
	15	0.863 ± 0.072^b^	0.251 ± 0.035^c^	1.369 ± 0.029^b*^	3.814 ± 0.169^a^	0.548 ± 0.050^b*^	0.647 ± 0.067^a^	0.654 ± 0.018^b*^
*Cyperus longus*	5	14.690 ± 2.280^a^	0.312 ± 0.041^a^	0.374 ± 0.035^b^	2.105 ± 0.145^b^	0.243 ± 0.022^a^	0.334 ± 0.057^b^	0.432 ± 0.051^a^
	10	6.248 ± 0.491^b^	0.174 ± 0.015^b^	0.676 ± 0.072^a^	2.145 ± 0.149^b^	0.191 ± 0.014^b^	0.554 ± 0.098^a^	0.249 ± 0.028^b^
	15	1.490 ± 0.054^c*^	0.205 ± 0.029^a^	0.726 ± 0.017^a^	3.897 ± 0.179^a^	0.281 ± 0.052^a^	0.490 ± 0.059^a^	0.378 ± 0.005^b^
*Cyperus rotundus*	5	3.012 ± 0.780^a^	0.291 ± 0.042^a^	1.428 ± 0.732^a^	0.974 ± 0.047^a^	0.360 ± 0.005^a^	0.498 ± 0.036^b^	0.533 ± 0.029^a^
	10	4.541 ± 0.659^a^	0.275 ± 0.098^a^	0.536 ± 0.114^b^	0.802 ± 0.017^a^	0.289 ± 0.056^a^	0.886 ± 0.077^a^	0.331 ± 0.130^a^
	15	1.023 ± 0.192^b^	0.249 ± 0.108^a^	1.219 ± 0.588^a*^	1.022 ± 0.221^a^	0.193 ± 0.025^b^	0.714 ± 0.137^a^	0.266 ± 0.084^b^
*Iris pseudacorus*	5	0.102 ± 0.022^b^	0.137 ± 0.017^a^	1.292 ± 0.419^a^	0.047 ± 0.015^b^	0.245 ± 0.013^b^	0.028 ± 0.002^b^	0.397 ± 0.186^c^
	10	1.085 ± 0.176^a^	0.148 ± 0.065^a^	0.994 ± 0.349^ab^	0.354 ± 0.224^a^	0.809 ± 0.076^a^	0.051 ± 0.002^a^	1.193 ± 0.139^a*^
	15	0.861 ± 0.208^a^	0.381 ± 0.144^b*^	0.383 ± 0.066^b^	0.274 ± 0.034^a^	0.303 ± 0.066^b^	0.043 ± 0.004^a^	0.676 ± 0.154^b*^
*Juncus effusus*	5	0.908 ± 0.239^a^	0.031 ± 0.006^b^	0.101 ± 0.024^b^	2.169 ± 0.599^a^	0.093 ± 0.016^a^	0.297 ± 0.018^b^	0.122 ± 0.004^b^
	10	0.374 ± 0.100^b^	0.079 ± 0.016^ab^	0.123 ± 0.012^b^	2.649 ± 0.430^a^	0.108 ± 0.004^a^	0.446 ± 0.037^ab^	0.190 ± 0.032^a^
	15	0.385 ± 0.021^b^	0.102 ± 0.041^a^	0.197 ± 0.019^a^	0.882 ± 0.058^b^	0.105 ± 0.005^a^	0.645 ± 0.035^a^	0.184 ± 0.009^a^
*Lythrum salicaria*	5	0.136 ± 0.040^b^	0.036 ± 0.024^b^	0.070 ± 0.030^b^	0.334 ± 0.010^b^	0.077 ± 0.023^a^	0.218 ± 0.020^b^	0.151 ± 0.040^b^
	10	0.449 ± 0.118^a^	0.053 ± 0.001^b^	0.093 ± 0.010^b^	0.683 ± 0.074^a^	0.051 ± 0.002^b^	0.611 ± 0.163^a^	0.207 ± 0.010^a^
	15	0.372 ± 0.040^a^	0.117 ± 0.054^a^	0.242 ± 0.013^a^	0.604 ± 0.100^a^	0.093 ± 0.019^a^	0.660 ± 0.022^a^	0.208 ± 0.047^a^
*Mentha aquatica*	5	3.290 ± 0.614^a^	0.441 ± 0.032^a^	2.832 ± 0.410^a^	1.412 ± 0.163^c^	0.420 ± 0.021^a^	0.544 ± 0.031^b^	0.545 ± 0.043^a^
	10	4.555 ± 0.098^a^	0.206 ± 0.028^b^	0.859 ± 0.146^b^	2.020 ± 0.189^b^	0.144 ± 0.006^b^	1.032 ± 0.162^a^	0.195 ± 0.014^b^
	15	0.934 ± 0.060^b^	0.138 ± 0.018^c^	0.772 ± 0.085^b^	4.918 ± 0.587^a*^	0.188 ± 0.022^b^	1.513 ± 0.142^a^	0.189 ± 0.019^b^
*Phragmites australis*	5	13.720 ± 0.874^a^	0.247 ± 0.007^a^	0.281 ± 0.030^a^	0.521 ± 0.070^b^	0.173 ± 0.010^b^	0.225 ± 0.015^b^	0.256 ± 0.019^b^
	10	11.369 ± 1.494^a^	0.226 ± 0.029^a^	0.370 ± 0.045^a^	0.953 ± 0.035^a^	0.257 ± 0.042^a^	0.562 ± 0.095^a^	0.432 ± 0.003^a^
	15	1.586 ± 0.165^b*^	0.239 ± 0.024^a^	0.111 ± 0.006^b^	0.977 ± 0.115^a^	0.208 ± 0.031^a^	0.415 ± 0.098^a^	0.240 ± 0.010^b^
*Scirpus holoschoenus*	5	3.746 ± 0.656^a^	0.366 ± 0.081^a^	0.675 ± 0.064^a^	0.807 ± 0.116^b^	0.275 ± 0.021^b^	2.159 ± 0.175^b*^	0.361 ± 0.007^b^
	10	4.064 ± 0.186^a^	0.350 ± 0.046^a^	0.603 ± 0.064^a^	1.417 ± 0.032^b^	0.436 ± 0.040^a^	2.727 ± 0.108^b*^	0.477 ± 0.009^a^
	15	1.455 ± 0.207^b*^	0.306 ± 0.036^a*^	0.497 ± 0.006^b^	3.080 ± 0.338^a^	0.476 ± 0.034^a*^	3.222 ± 0.082^a*^	0.397 ± 0.040^a^
*Typha angustifolia*	5	5.749 ± 1.131^b^	0.536 ± 0.054^ab^	1.725 ± 0.087^b^	0.342 ± 0.027^b^	0.483 ± 0.025^b^	0.466 ± 0.027^b^	0.588 ± 0.084^b^
	10	13.692 ± 2.020^a*^	0.825 ± 0.018^a^	3.441 ± 0.376^a*^	0.583 ± 0.025^a^	0.855 ± 0.036^a*^	1.313 ± 0.073^a^	0.986 ± 0.046^a^
	15	0.455 ± 0.051^c^	0.290 ± 0.053^b*^	0.844 ± 0.094^c^	0.696 ± 0.044^a^	0.444 ± 0.012^b*^	1.260 ± 0.179^a^	0.451 ± 0.071^b^

^+^Days of Exposure. In super script alphabets indicate significant difference between bioconcentration factor of the different studied plants with “a” being highest followed by later alphabets. Data is presented in Mean ± SD, with *n* = 3, at *p* < 0.05. Asterisk (*) on bars indicates significantly highest metal(loid) uptake for each corresponding metal on specified day (5, 10, or 15th)

The translocation factors (TF) for each metal(loid) and plant on days 5, 10, and 15 are presented in Table 5. *L. salicaria* exhibited the highest TF for As on the 5th (2.5) and 10th (0.2) day, while *M. aquatica* showed the highest TF (0.2) on day 15. For Cd, *J. effusus*, showed an increasing TF trend as time progressed with 1.4 on day 10 and 2.2 on day 15. The highest TF for Cu were also observed for *J. effusus* with 0.8 on day 10 and 0.7 on day 15, whereas *C. rotundus* showed the highest TFs (~ 0.7) for Fe on days 10 and 15. *J. effusus* also showed the highest TF for Ni (2.5) on day 15. For Pb *P. australis* showed the highest TF at all exposure intervals with 3.9 on day 5, 1.5 on day 10 and 1.8 on day 15. For Zn *I. pseudacorus* (1.2) and *L. salicaria* showed the greatest TF on day 5, whereas *T. angustifolia* (1.1) showed the largest TFs on day 10 and 15.

**Table 5:** Translocation factors (TF) of the studied wetland plants after 5, 10, and 15 days exposure to metal(loid)s polluted groundwater

Plant	Days^+^	As	Cd	Cu	Fe	Ni	Pb	Zn
*Carex riparia*	5	0.082 ± 0.005^a^	0.318 ± 0.033^a^	0.106 ± 0.005^a^	0.141 ± 0.014^a^	0.343 ± 0.012^b^	0.508 ± 0.032^a^	0.375 ± 0.030^a^
	10	0.067 ± 0.003^b^	0.230 ± 0.024^b^	0.138 ± 0.009^a^	0.086 ± 0.002^ab^	0.713 ± 0.076^a^	0.472 ± 0.029^a^	0.353 ± 0.014^a^
	15	0.058 ± 0.005^b^	0.299 ± 0.018^ab^	0.069 ± 0.002^b^	0.062 ± 0.007^b^	0.175 ± 0.011^c^	0.452 ± 0.056^b^	0.262 ± 0.028^b^
*Cyperus longus*	5	0.007 ± 0.000^a^	0.372 ± 0.085^b^	0.054 ± 0.003^b^	0.030 ± 0.003^a^	0.374 ± 0.020^b^	0.877 ± 0.075^a^	0.441 ± 0.210^a^
	10	0.007 ± 0.000^a^	0.489 ± 0.048^a^	0.119 ± 0.003^a^	0.032 ± 0.001^a^	0.552 ± 0.052^a^	0.356 ± 0.018^b^	0.594 ± 0.070^a^
	15	0.006 ± 0.000^a^	0.401 ± 0.015^ab^	0.125 ± 0.011^a^	0.018 ± 0.001^b^	0.203 ± 0.031^c^	0.229 ± 0.025^b^	0.259 ± 0.002^b^
*Cyperus rotundus*	5	0.149 ± 0.029^a^	0.539 ± 0.014^a^	0.199 ± 0.015^a^	0.367 ± 0.046^b^	0.640 ± 0.051^a^	0.491 ± 0.036^a^	0.499 ± 0.054^b^
	10	0.106 ± 0.015^b^	0.263 ± 0.060^b^	0.184 ± 0.011^a^	0.703 ± 0.033^a*^	0.311 ± 0.034^b^	0.455 ± 0.063^a^	0.620 ± 0.035^a^
	15	0.120 ± 0.007^b^	0.245 ± 0.043^b^	0.156 ± 0.005^b^	0.676 ± 0.014^a*^	0.216 ± 0.016^c^	0.499 ± 0.056^a^	0.708 ± 0.171^a^
*Iris pseudacorus*	5	0.144 ± 0.038^a^	0.820 ± 0.039^a^	0.627 ± 0.224^a^	0.043 ± 0.011^a^	0.034 ± 0.004^a^	0.631 ± 0.041^a^	1.203 ± 0.522^a^
	10	0.034 ± 0.004^b^	0.074 ± 0.027^a^	0.586 ± 0.097^a^	0.031 ± 0.016^a^	0.002 ± 0.000^a^	0.385 ± 0.028^b^	0.419 ± 0.069^b^
	15	0.061 ± 0.018^b^	0.014 ± 0.005^b^	0.415 ± 0.090^b^	0.042 ± 0.005^a^	0.029 ± 0.004^a^	0.543 ± 0.046^a^	0.214 ± 0.059^b^
*Juncus effusus*	5	0.154 ± 0.014^a^	1.654 ± 0.720^ab^	0.440 ± 0.076^b^	0.073 ± 0.024^c^	0.448 ± 0.032^c^	0.313 ± 0.009^a^	0.715 ± 0.063^a^
	10	0.210 ± 0.034^a^	1.371 ± 0.499^b*^	0.834 ± 0.003^a*^	0.164 ± 0.013^b^	1.305 ± 0.230^b^	0.308 ± 0.019^a^	1.175 ± 0.163^b*^
	15	0.070 ± 0.010^b^	2.166 ± 1.105^a*^	0.702 ± 0.091^a*^	0.241 ± 0.015^a^	2.527 ± 0.120^a*^	0.241 ± 0.014^b^	2.235 ± 0.174^c*^
*Lythrum salicaria*	5	2.548 ± 0.989^a*^	2.735 ± 2.496^a*^	2.083 ± 1.072^a*^	0.293 ± 0.003^a^	0.557 ± 0.180^b^	0.341 ± 0.026^a^	2.739 ± 0.560^a*^
	10	0.245 ± 0.033^b*^	0.124 ± 0.000^c^	0.384 ± 0.034^b^	0.204 ± 0.011^b^	0.233 ± 0.031^c^	0.115 ± 0.013^b^	0.808 ± 0.062^b^
	15	0.199 ± 0.037^b^	0.409 ± 0.180^b^	0.269 ± 0.024^b^	0.267 ± 0.048^ab^	0.791 ± 0.149^a^	0.109 ± 0.007^b^	0.658 ± 0.237^b^
*Mentha aquatica*	5	0.310 ± 0.032^a^	1.072 ± 0.148^a^	0.149 ± 0.018^a^	0.450 ± 0.049^a*^	1.206 ± 0.011^b*^	0.700 ± 0.088^a^	1.026 ± 0.050^a^
	10	0.168 ± 0.005^b^	0.698 ± 0.157^b^	0.116 ± 0.006^ab^	0.172 ± 0.022^b^	1.644 ± 0.090^a*^	0.358 ± 0.089^b^	0.958 ± 0.053^ab^
	15	0.242 ± 0.018^ab*^	0.436 ± 0.083^c^	0.091 ± 0.008^b^	0.047 ± 0.007^c^	0.540 ± 0.046^c^	0.207 ± 0.014^b^	0.665 ± 0.021^a^
*Phragmites australis*	5	0.094 ± 0.008^b^	0.016 ± 0.002^a^	0.371 ± 0.050^ab^	0.431 ± 0.044^a*^	0.232 ± 0.001^a^	3.916 ± 0.264^a*^	0.928 ± 0.144^a^
	10	0.095 ± 0.008^b^	0.009 ± 0.001^b^	0.260 ± 0.008^a^	0.319 ± 0.009^b^	0.150 ± 0.015^b^	1.539 ± 0.092^b*^	0.578 ± 0.044^b^
	15	0.161 ± 0.004^a^	0.005 ± 0.000^b^	0.453 ± 0.018^a^	0.451 ± 0.057^a^	0.078 ± 0.017^c^	1.878 ± 0.734^b*^	0.523 ± 0.015^b^
*Scirpus holoschoenus*	5	0.081 ± 0.017^a^	0.223 ± 0.026^a^	0.140 ± 0.017^a^	0.342 ± 0.056^a^	0.308 ± 0.077^a^	0.220 ± 0.013^a^	0.468 ± 0.024^a^
	10	0.080 ± 0.006^a^	0.136 ± 0.004^b^	0.072 ± 0.027^b^	0.207 ± 0.011^b^	0.099 ± 0.012^b^	0.141 ± 0.004^b^	0.293 ± 0.016^b^
	15	0.055 ± 0.006^b^	0.122 ± 0.007^b^	0.104 ± 0.014^ab^	0.178 ± 0.018^c^	0.099 ± 0.004^b^	0.119 ± 0.017^b^	0.358 ± 0.037^b^
*Typha angustifolia*	5	0.026 ± 0.003^b^	0.521 ± 0.118^b^	0.408 ± 0.029^a^	0.206 ± 0.034^a^	1.338 ± 0.103^a*^	2.489 ± 0.086^a^	0.863 ± 0.039^b^
	10	0.044 ± 0.008^a^	1.461 ± 0.101^a*^	0.261 ± 0.009^b^	0.261 ± 0.018^a^	1.704 ± 0.058^a*^	0.411 ± 0.036^b^	1.068 ± 0.002^a*^
	15	0.036 ± 0.004^ab^	0.953 ± 0.057^b^	0.568 ± 0.046^a^	0.490 ± 0.062^b^	0.594 ± 0.021^b^	0.303 ± 0.020^b^	1.099 ± 0.181^a^

^+^Days of Exposure. In super script alphabets indicate significant difference between translocation factor of the different studied plants with “a” being highest followed by later alphabets. Data is presented in Mean ± SD, with *n* = 3, at *p* < 0.05. Asterisk (*) on bars indicates significantly highest metal(loid) uptake for each corresponding metal on specified day (5, 10, or 15th)

## Discussion

Phytoremediation can be a sustainable approach to treat contaminated media such as soil and water (Khan et al. 2023). However, finding plants that tolerate high contaminant concentrations, as is often the case at polluted sites is not obvious. Macrophytes are among the most effective plants to tolerate and remove metal(loid)s in aquatic environments (Rai 2019). As a result of high metal(loid)s levels in the water, plants’ physiology can be easily altered in many species. Physiological alteration refers to immediate, non-inherited changes in plants under metal stress, like reduced growth or disrupted enzyme activity. These are the plant's coping mechanisms, not true adaptation. This can be observed after short periods of exposure as high metal(loid)s uptake in roots occur leading to interbody sequestration (Sricoth et al. 2018). For phyto-management of both legacy and emerging pollutants a wide variety of aquatic plant types are potentially interesting including submergent, emergent, and floating species (Rezania et al. 2021; Hussain et al. 2021). Aquatic species can be potentially cultivated in constructed or semi-natural wetland systems to enhance metal(loid) removal and improve site aesthetic and ecological characteristics (Guo et al. 2020). However, the potential of each plant needs to be assessed carefully since each plant genotype and ecotype can display a variable level of metal(loid) tolerance and uptake ability (Khan et al. 2021a, b).

In the present study, a wide variety of responses was observed for the 10 macrophyte species studied including no impact versus significant changes in plant height and biomass (increase or decrease) when exposed to contaminated water (Fig. 1, Table 1). Plant height is reported as indicator of metal(loid)s tolerance (El-Meihy et al. 2019). In the current study no significant changes in plant height on 15th days were noted for *C. riparia*, *C. longus*, *C. rotundus*, *J. effusus, M. aquatica*, *S. holoschoenus*, and *T. angustifolia.* The plant height at the interval of 5 days revealed that *C. longus, C. rotundus*, *J. effusus*, *L. salicaria*, and *T. angustifolia* showed stunted growth caused by the acute metal(loid)s exposure (Fig. 1b). This observation suggests a degree of resilience in these plants. While plants often exhibit resilience mechanisms in response to metal(loid) stress, recovery doesn't always imply a complete return to pre-stress physiological states. In this case, the observed height recovery suggests a potential for improvement in certain growth attributes, but it’s important to note that underlying physiological processes (photosynthesis, stomatal conductance, generation of reactive oxygen species, carbon fixation, and transpiration rates) might still be affected (Arshad et al. 2017). Similarly, plant biomass is also a good indicator of metal(loid) tolerance. In the present study, various macrophytes produced 1.2- to 2.1-time greater biomass on a fresh basis including *S. holoschoenus*, *P. australis*, *C. rotundus*, *I. pseudacorus*, *L. salicaria*, *M. aquatica* when exposed to contaminated water (Table 1). Similar results (1.3 to 6 times more biomass) were observed when biomass was expressed on a dry weight basis for *C. longus*, *I. pseudacorus*, *M. aquatica*, *T. angustifolia*, *C. riparia*, *J. effusus*, *C. rotundus*, *S. holoschoenus*, and *L. salicaria*. Schück and Greger (2020b) also found increased biomass in macrophytes exposed to contaminated water and reported that for 34 wetland plants, a 38-fold difference in total biomass was noted between the highest biomass producer *Dryopteris carthusiana* and the lowest producer *Eriophorum angustifolium*. Sricoth et al. (2018) also observed differences between the biomass produced by plants (*Thalia geniculate*, *Cyperus alternifolius*, *Canna indica*, *Eichhornia crassipes*, *Pistia stratiotes*) exposed to different concentrations of Zn (10, 20, and 40 mg L^−1^) and Cd (2, 4, and 8 mg L^−1^). As observed in the present study (Supplementary Fig. 11 and Supplementary Fig. 12), while metal(loid)s polluted water can lead to a puzzling increase in a plant’s fresh weight and other physical parameters, due to increased water uptake for dilution, the plant’s dry weight, representing actual growth, may decrease (Wang et al. 2020b). This can happen because the pollutants hinder the plant’s ability to utilize water and nutrients effectively, or because the plant prioritizes stress response and defence mechanisms overgrowth, leading to water storage instead of tissue production (Ofori et al. 2021). Additionally, the pollution might directly affect the plant’s cellular composition, causing an accumulation of water-filled vacuoles that contribute to weight but not growth (Khan et al. 2021a, b). To fully understand the extent of recovery and the long-term effects on growth, further research could explore specific physiological parameters like photosynthesis, respiration, stomatal conductance, and ROS generation, upon exposure to the pollutants.

Significant differences in the metal(loid) levels were observed in the roots and shoots of the studied plants. In other words, the uptake of metal(loid)s depends on the capacity of plant species, geno/ecotype of the plant (capacity to convert metal into less toxic forms by vascular compartmentalization), and the concentration of the contaminants in the water (Schück and Greger 2020a; Sricoth et al. 2018). In a multi-metal(loid) contaminated condition, it is challenging to evaluate the impacts of an individual metal(loid) on a specific plant compartment over time. In the present study, these impacts were analysed using a constructed MLR based model in which plant dried biomass (shoot or root) was considered as dependent variable and the concentration of metal(loid)s in plant dried biomass (root or shoot) and exposure time as independent variables. A metal uptake, as a variable accounting for higher total model variability, is considered as an important factor to plant biomass in the present study. This type of analysis can help to identify the most important CPCs for plant performance when multiple variables are considered.

The bioaccumulation factor (BAF) is indicative of “above-ground” metal(loid) storage—metal(loid) transfer from the soil or sediment to the shoots, whereas the bioconcentration factor (BCF) is representative of “below-ground” storage -metal(loid) transfer from the soil/sediment to the roots (Raza et al. 2019; Aftab et al. 2021). In the present study, both BAF and BCF were generally less than 1 at the end of the 15 days exposure time. The TF or shoot:root ratio was also less than 1 suggesting greater metal(loid) accumulation in “below-ground” biomass rather than in “above-ground” biomass. Altogether these data indicate an “excluder” strategy for the macrophytes investigated and their potential for phytostabilization rather than phytoextraction approaches (Wang et al. 2020a, b; Qurban et al. 2021). The fact that metal(loid) concentrations in the rhizospheric soil increased as exposure time progressed and were significantly higher than those measured in the absence of plants for the same time interval, is clearly indicative of active phytostabilization processes by the macrophytes in the rhizosphere. It should be noted that the macrophytes investigated are anemophilous species and not commonly grazed by animals. As a result, bioaccumulation via the food chain is not relevant for these plants. Yet, accumulation in shoots decreases as a rule with subsequent harvests. Hence, cropping of the macrophytes for a few cycles could minimize metal(loid) cycling as leaves fall off and decay. It should be noted that the present study was conducted under batch conditions and, therefore, does not address situations in which polluted water continuously enters the system. It is, thus, representative of scenarios for which the contamination source has been stopped or eliminated and no additional contaminant inputs occur.

Phytoremediation generally enjoys the status of being a green and eco-friendly method for the treatment of contaminated soils, sediments and/or waters (Yousaf et al. 2022). However, the technical and economic potential of phytoremediation and the specific phytoremediation strategy (e.g., stabilization, accumulation, extraction, etc.) need to be carefully assessed for every site or environment (Qurban et al. 2021). Identifying the suitable plants via treatability testing at lab scale is an important step in validating a specific phytoremediation approach for a target site (Nawaz et al. 2024; Khan et al. 2021b). This is particularly relevant for aquatic environments for which experience on phytoremediation and phyto-restoration in long-term and large-scale projects is scarce. The approach followed in this study can be extended to other types of polluted waters and macrophytes. The potential of the best performing macrophytes in this study will be assessed further in a constructed wetland system as part of another study at mesoscale. Even though the studied wetland plants showed a higher uptake in the roots than in the shoots, these plants are emergent and are easy to harvest. Hence, metal(loid)s may be removed further from the water with additional biomass harvests (Khan et al. 2021a; Schück and Greger 2020a, b). In addition, the exposure time plays a significant role in the removal of the metal(loid); the higher the hydraulic retention time within the wetland system, the greater the time plants get to remove the contaminants (Minakshi et al. 2022; Wang et al. 2021). For plants characterized by a low root to shoot transfer, intercropping with accumulator or hyperaccumulator plants may be an interesting option (Sricoth et al. 2018). In this way, metal(loid)s stabilized in the rhizospheric region can be taken up more efficiently by accumulators/hyperaccumulators and, ultimately, improve removal from the system. The feasibility of such combined approaches needs to be carefully assessed for each site and contamination in advance as shown for the present study (Khan et al. 2023).

Further research should focus on directly analysing the composition of precipitates formed in the phytoremediation studies conducted at the microcosm, mesocosm, and macrocosm scale. Additionally, comparing metal speciation in the rhizosphere versus bulk soil will provide clearer evidence of plant-induced precipitation. Long-term studies would investigate the stability of these precipitates, while examining the influence of the rhizosphere microbiome could reveal additional factors shaping the precipitation process. Another opinion that would be interesting to exposure the identification of different type of metal(loid)s form present in the soil, during and after the phytoremediation study. This can be done using the sequential metal extraction, which can help analyse the different metal(loid)s distribution within different binding strength categories (exchangeable, carbonate-bound, Fe/Mn-oxide bound, organic matter bound, and residual) in the soil, indicating potential mobility and bioavailability, metal(loid)s species.

## Conclusion

The use of metal(loid) tolerant macrophytes for river margin stabilization and/or phytoremediation strategies is a nature-based approach with potential for buffering inputs from industrial or agricultural effluents. However, due consideration must be given to the selection of the plants for phytoremediation approaches. The results of this study suggest that native macrophytes differ significantly in their ability to tolerate and stabilize metal(loid)s under similar growing conditions. Some plants, particularly, *C. riparia*, *C. longus*, *C. rotundus*, *L. salicaria*, *I. pseudacorus*, *J. effusus*, and *M. aquatica*, can significantly uptake metal(loid)s into their biomass, but show symptoms of physiological stress, suggesting that they are unsuitable for long-term restoration or phytostabilization strategies. Other plants, such as *P. australis*, *S. holoschoenus*, and *T. angustifolia* showed lower uptake of metal(loid)s into the aerial parts and did not show symptoms of stress, indicating significant tolerance to metal(loid)s contamination and stabilization of metal(loid)s in the rhizosphere. As a result, these plants are potential candidates for phytostabilization and/or phyto-restoration strategies. In addition, our data suggest that the highest uptake per gram of biomass and acute impact on plants occurs during the initial exposure to the contaminant. In consequence, acclimatization of selected plants must be considered as an essential preliminary step in the phytoremediation/-restoration strategy.

## Supplementary Information

Below is the link to the electronic supplementary material.

Supplementary file1 (DOCX 28804 KB)

## Data Availability

Data and materials would be available on reasonable request.
