# Supplementary material for: Metal(loid) tolerance, accumulation, and phytoremediation potential of wetland macrophytes for multi-metal(loid)s polluted water
Source: Environ Sci Pollut Res Int. 2024 Nov 27;31(57):65724–40. doi: 10.1007/s11356-024-35519-5 (PMC11631999; doi:10.1007/s11356-024-35519-5)
Supplement: Supplementary file 1 — Supplementary file1 (DOCX 28804 KB) [file 11356_2024_35519_MOESM1_ESM.docx]

**Supplementary Material**

**Metal(loid) tolerance, accumulation, and phytoremediation potential of wetland macrophytes for multi-metal(loid)s polluted water.**

Aqib Hassan Ali Khan^a^, Blanca Velasco-Arroyo^b^, Carlos Rad^c^, Sandra Curiel-Alegre^ac^, Carlos Rumbo^a^, Herwig De Wilde^d^, Alfredo Pérez-de-Mora^e^, Sonia Martel-Martín^a^, Rocío Barros^a*^

*^a^International Research Center in Critical Raw Materials for Advanced Industrial Technologies (ICCRAM), University of Burgos, Centro de I+D+I. Plaza Misael Bañuelos s/n. 09001, Burgos, Spain*

*^b^Department of Biotechnology and Food Science, University of Burgos, Plaza Misael Bañuelos, s/n. 09001, Burgos, Spain*

*^c^Research Group in Composting (UBUCOMP), University of Burgos, Faculty of Sciences, Plaza Misael Bañuelos s/n, 09001, Burgos, Spain*

*^d^TAUW België nv, Dept. of Soil and Groundwater, Waaslandlaan 8A3, 9160 Lokeren, Belgium*

*^e^TAUW GmbH, Dept. of Soil and Groundwater, Landsbergerstr. 404, 81241 Munich, Germany*

***Corresponding authors:** [rbarros@ubu.es](mailto:rbarros@ubu.es)

Supplementary Table 1. Initial concentrations for the target elements (expressed in mg L^−1^) in the original groundwater extracted from different collected wells located at the polluted site.

| **Metal(loid)** | **mg L^−1^** |
| --- | --- |
| **As** | 0.300 – 0.500 |
| **Cd** | 2.000 – 2.500 |
| **Cu** | 160 – 1000 |
| **Fe** | 250 – 400 |
| **Ni** | 130 – 150 |
| **Pb** | 0.200 – 0.350 |
| **Zn** | 70 – 320 |


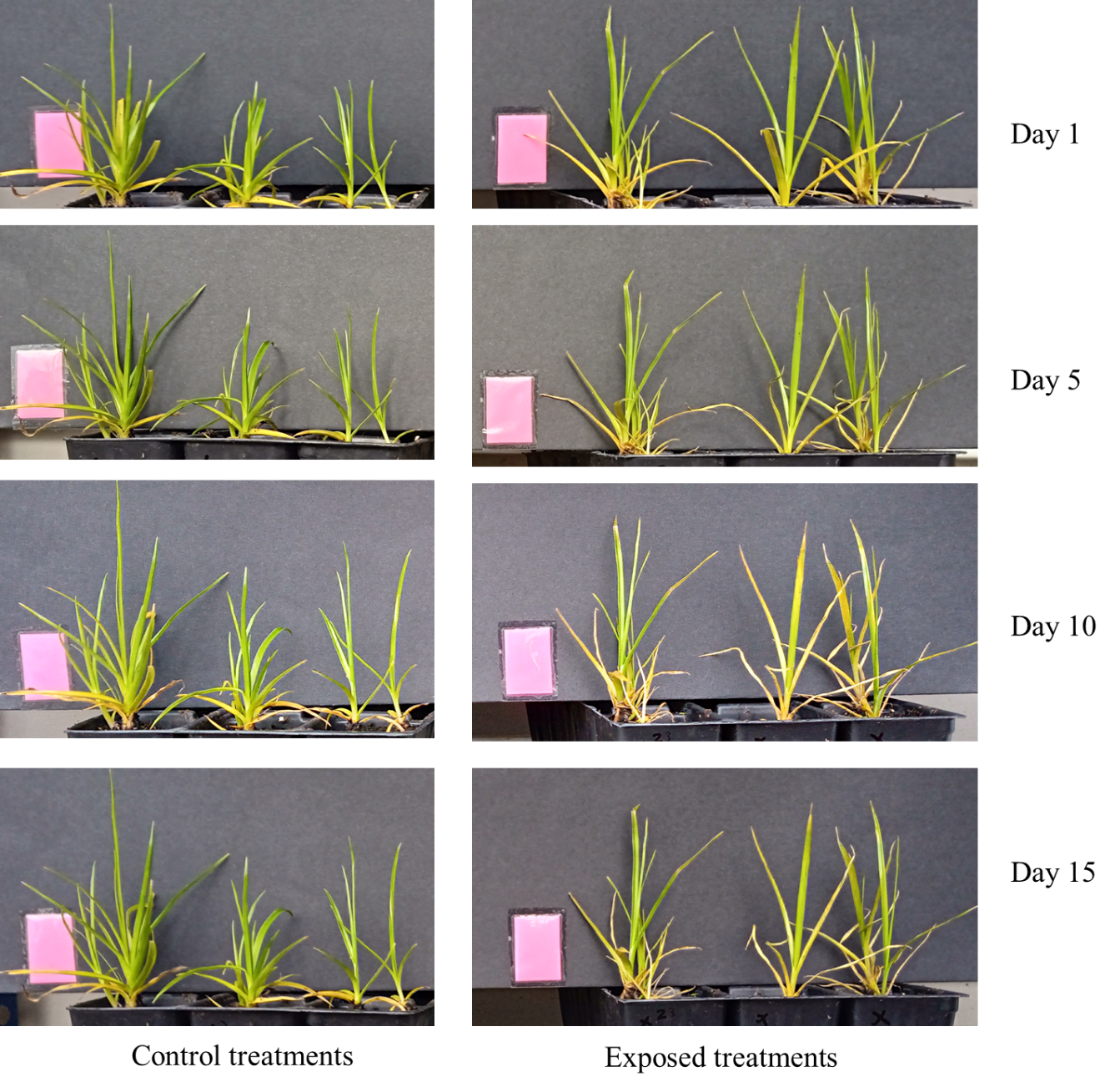


**Supplementry Figure 1.** Changes in growth of *Carex riparia* at 1, 5, 10, and 15 days.


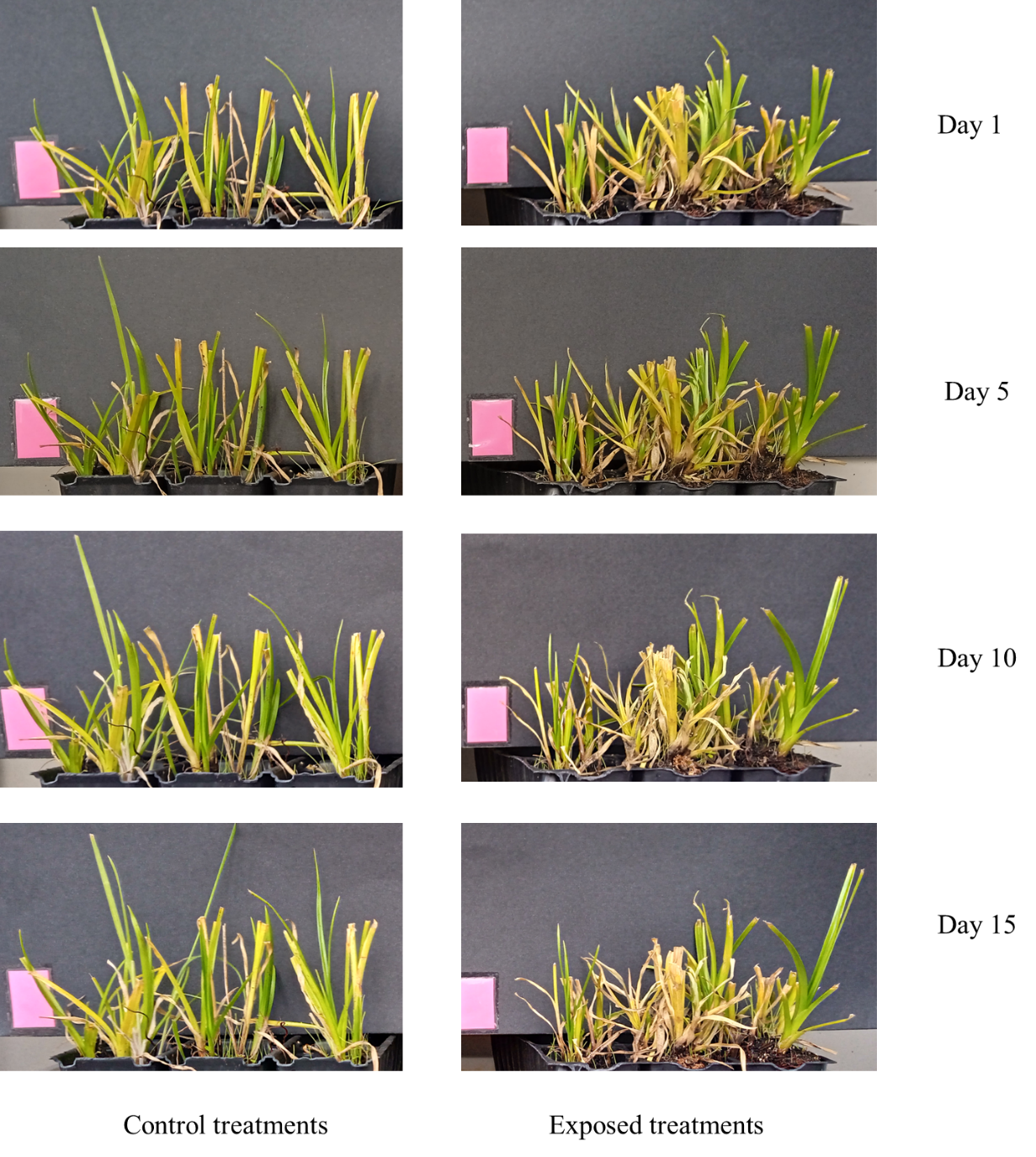


**Supplementry Figure 2.** Changes in growth of *Cyperus longus* at 1, 5, 10 and 15 days.


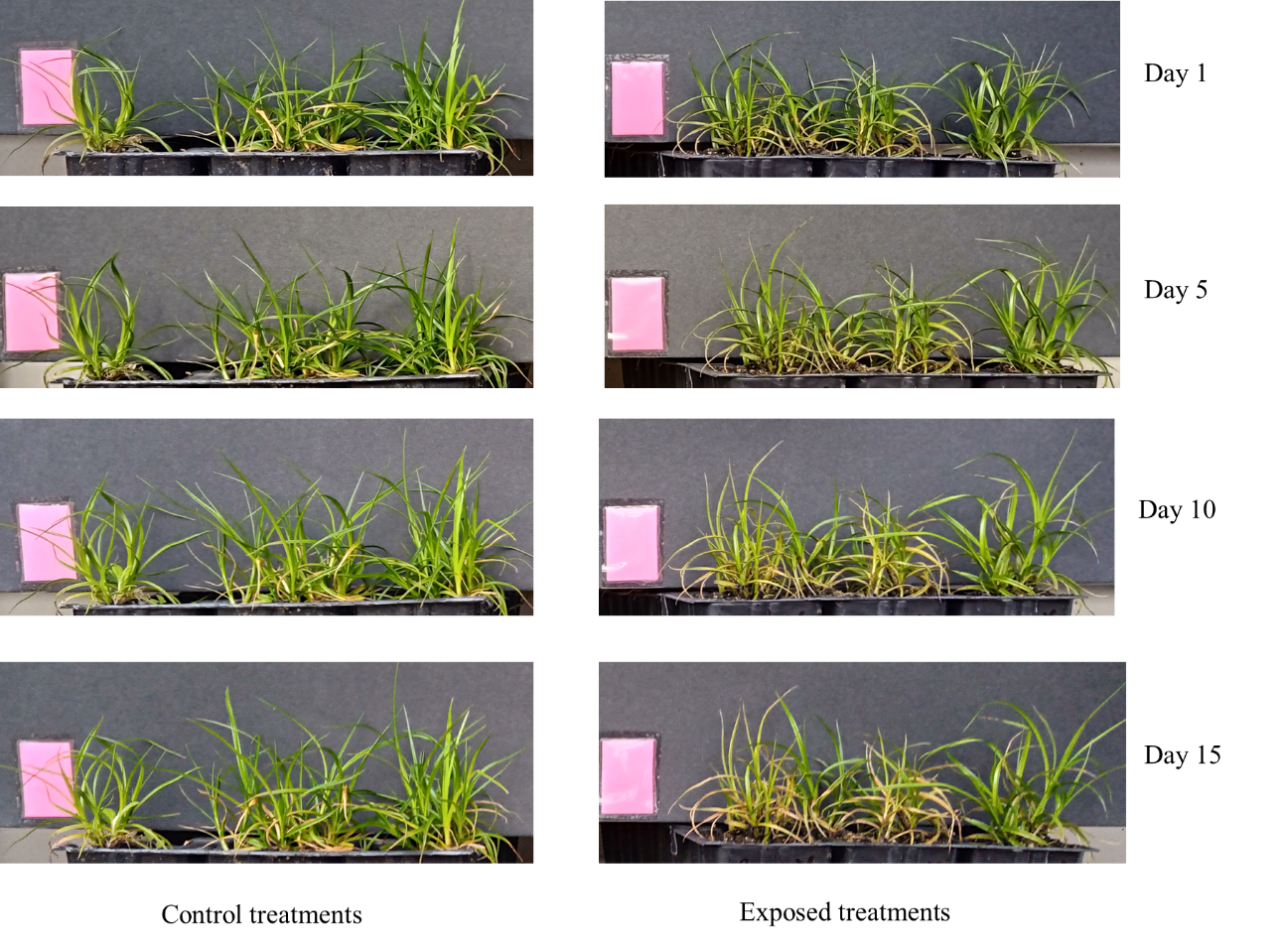


**Supplementry Figure 3.** Changes in growth of *Cyperus rotundus* at 1, 5, 10, and 15 days.


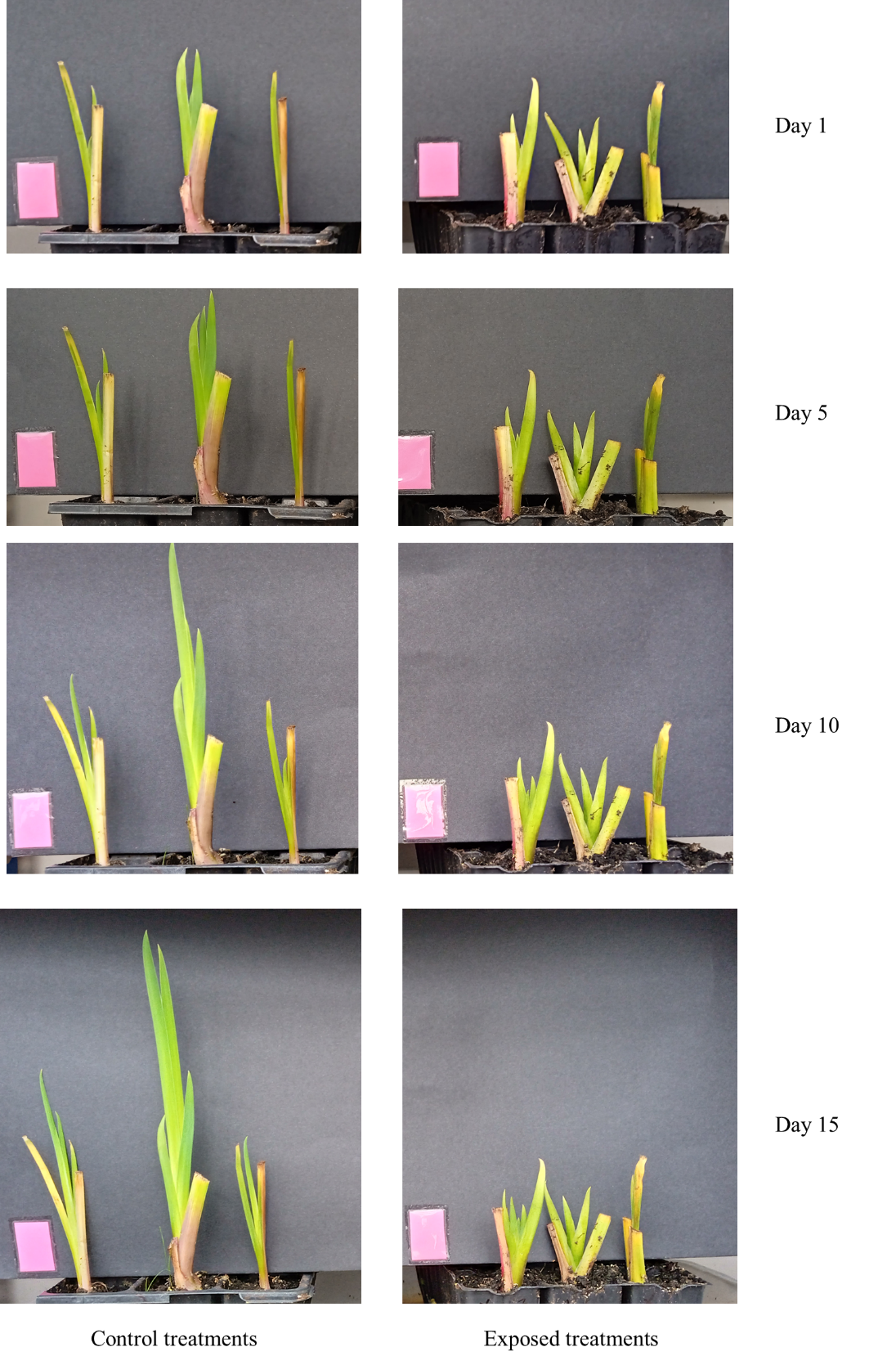


**Supplementry Figure 4.** Changes in growth of *Iris pseudacorus* at 0, 5, 10, and 15 days.


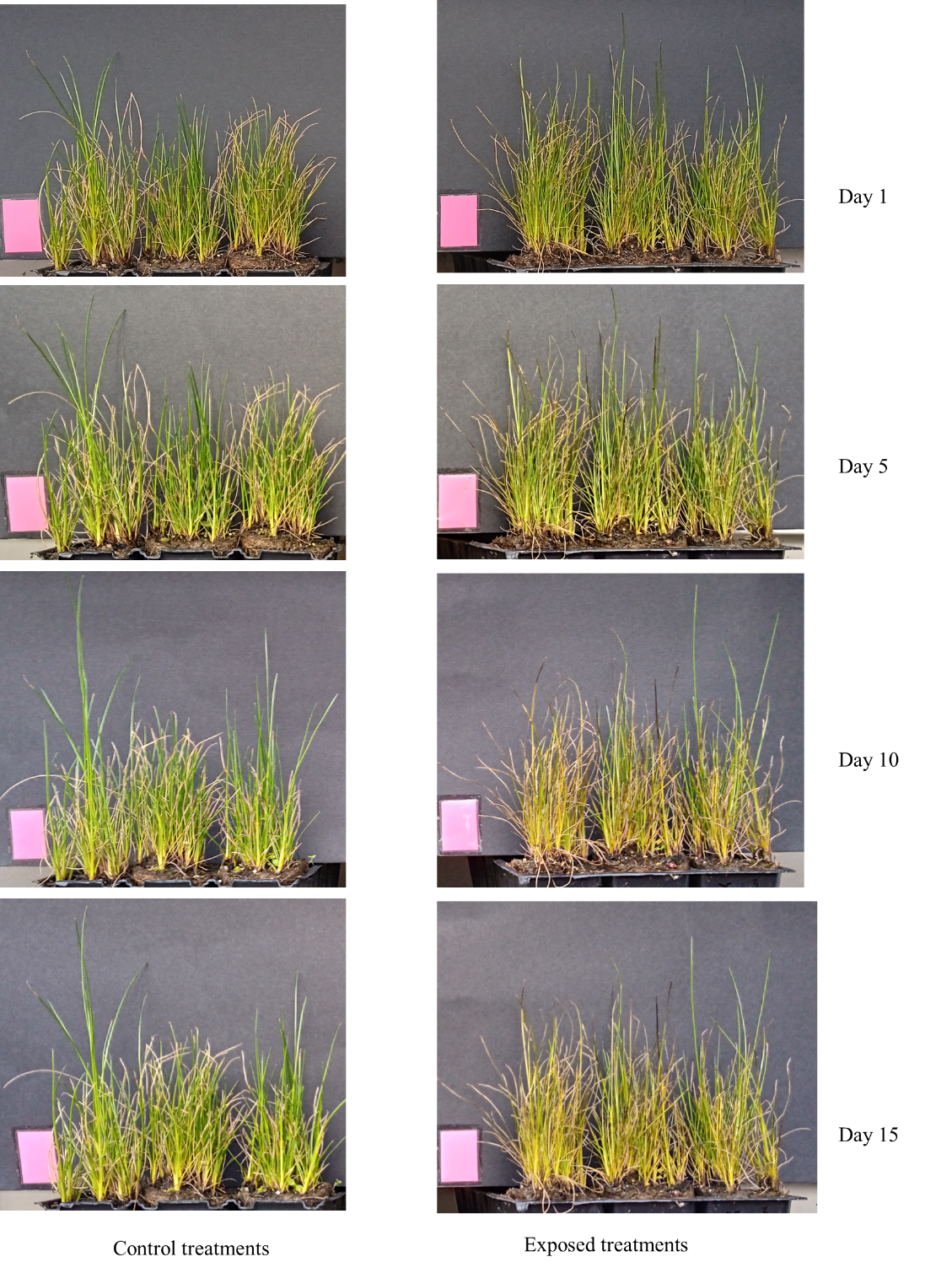


**Supplementry Figure 5.** Changes in growth of *Juncus effusus* at 1, 5, 10, and 15 days.


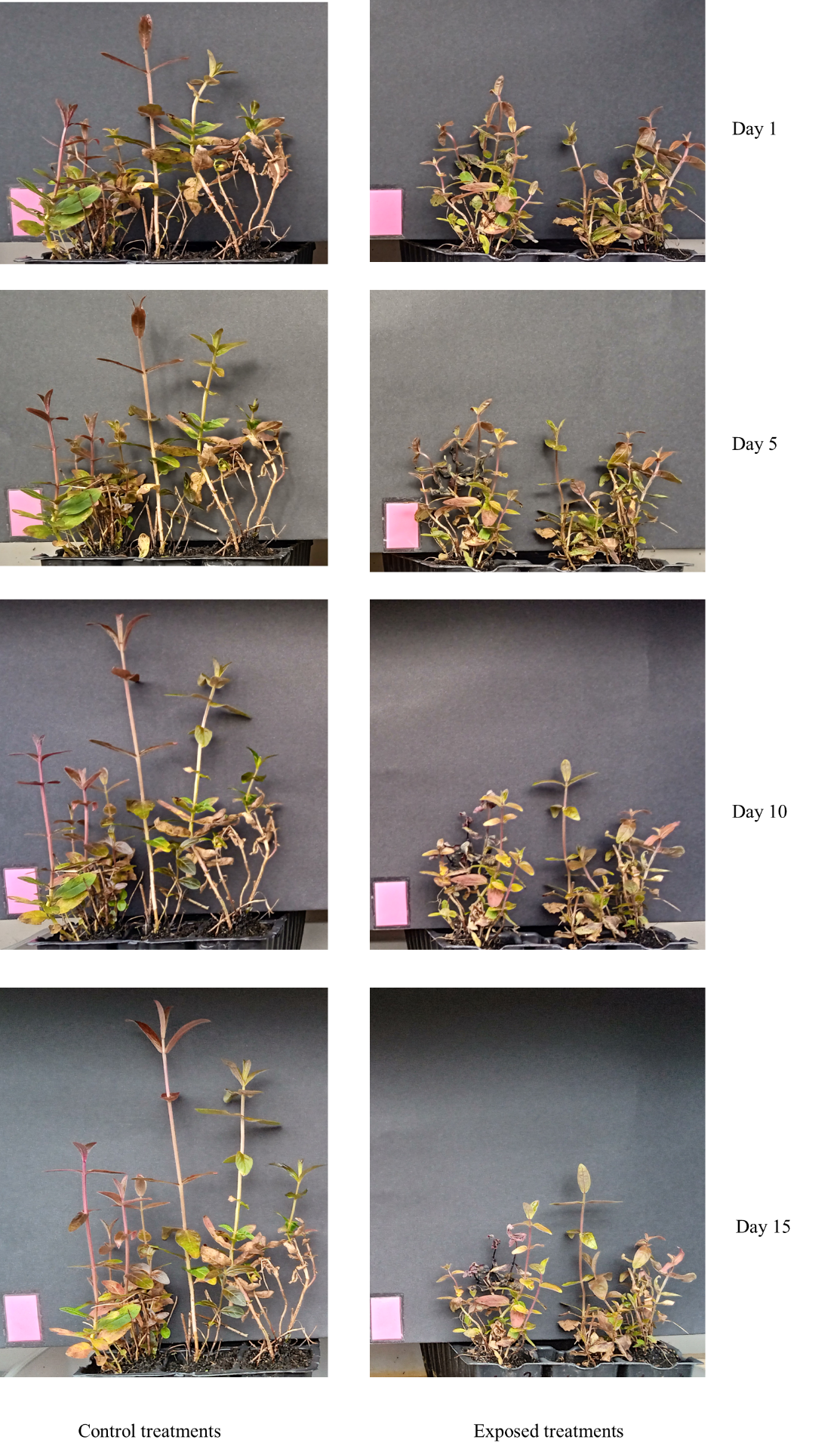


**Supplementry Figure 6.** Changes in growth of *Lythrum salicaria* at 1, 5, 10, and 15 days.


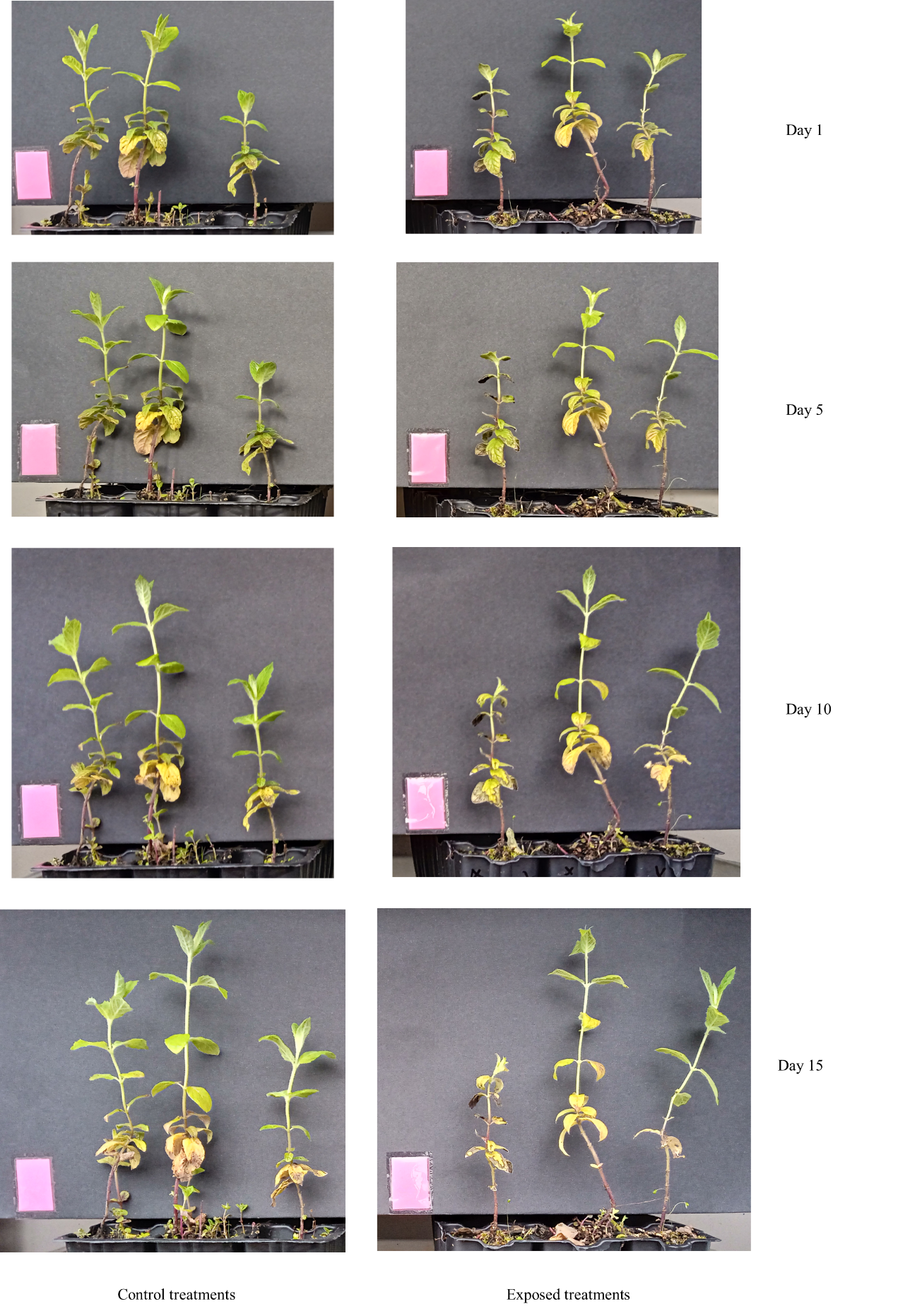


**Supplementry Figure 7.** Changes in growth of *Mentha aquatica* at 1, 5, 10, and 15 days.


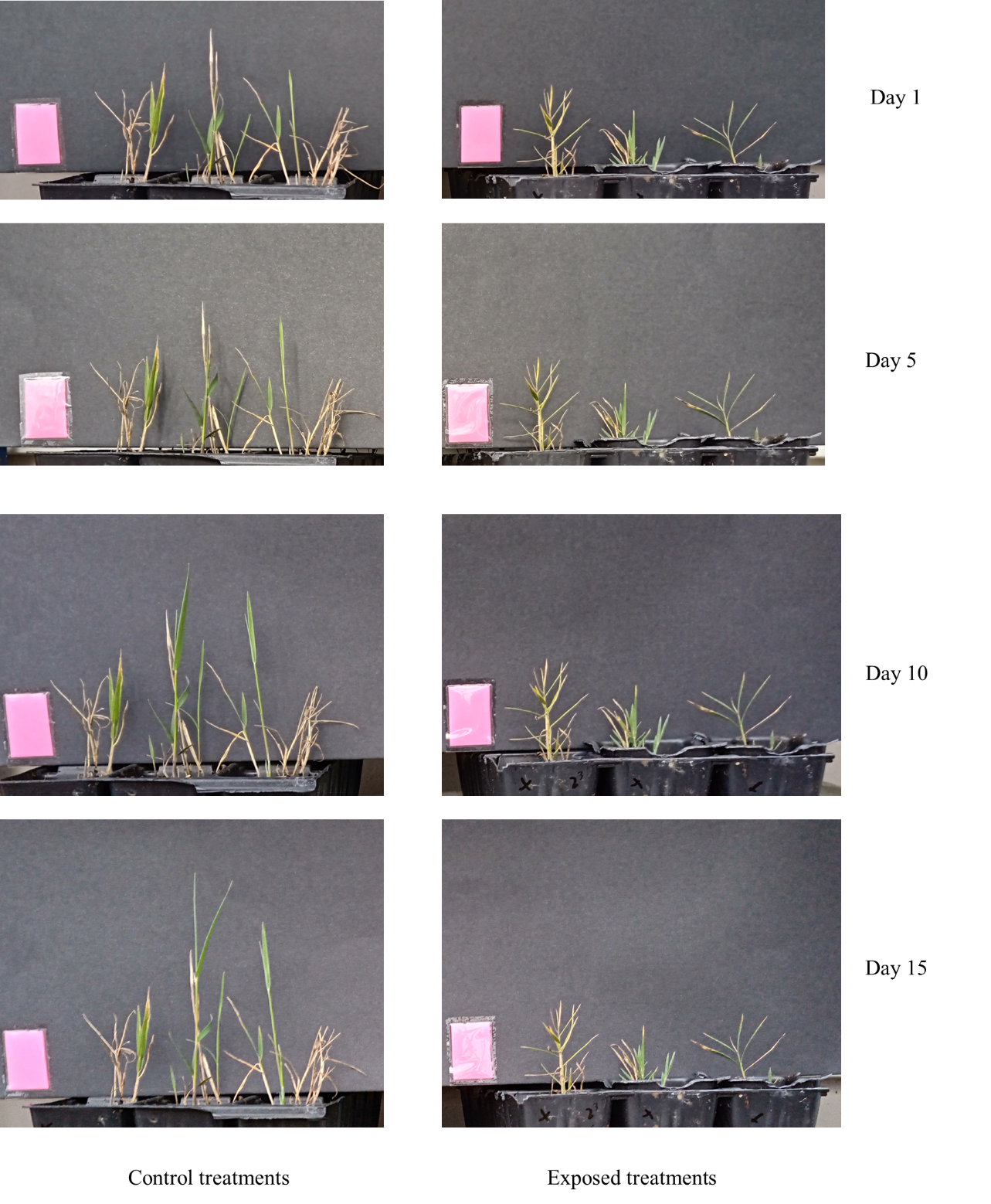


**Supplementry Figure 8.** Changes in growth of *Phragmites australis* at 1, 5, 10, and 15 days.


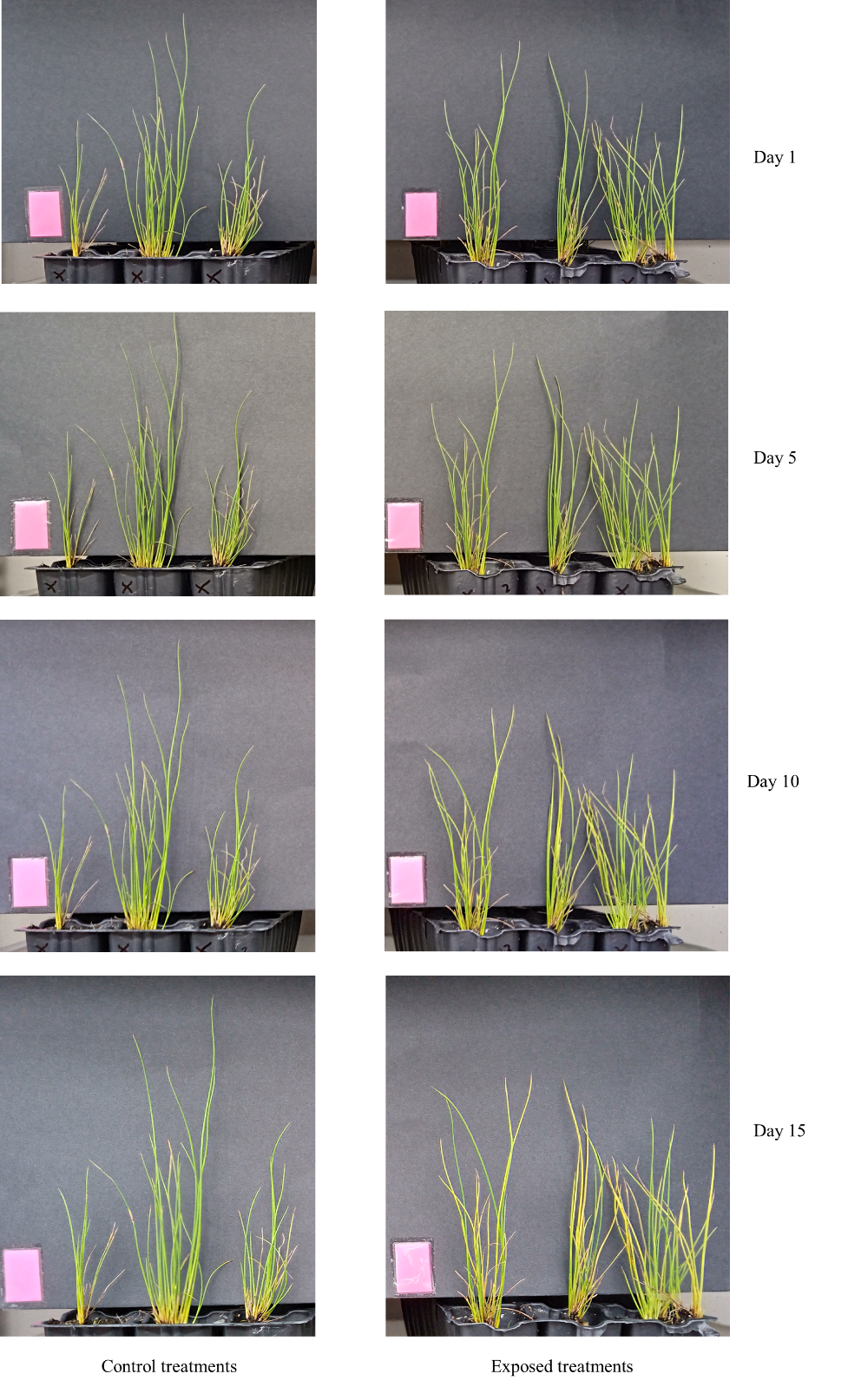


**Supplementry Figure 9.** Changes in growth of *Scirpus holoschoenus* at 1, 5, 10, and 15 days.


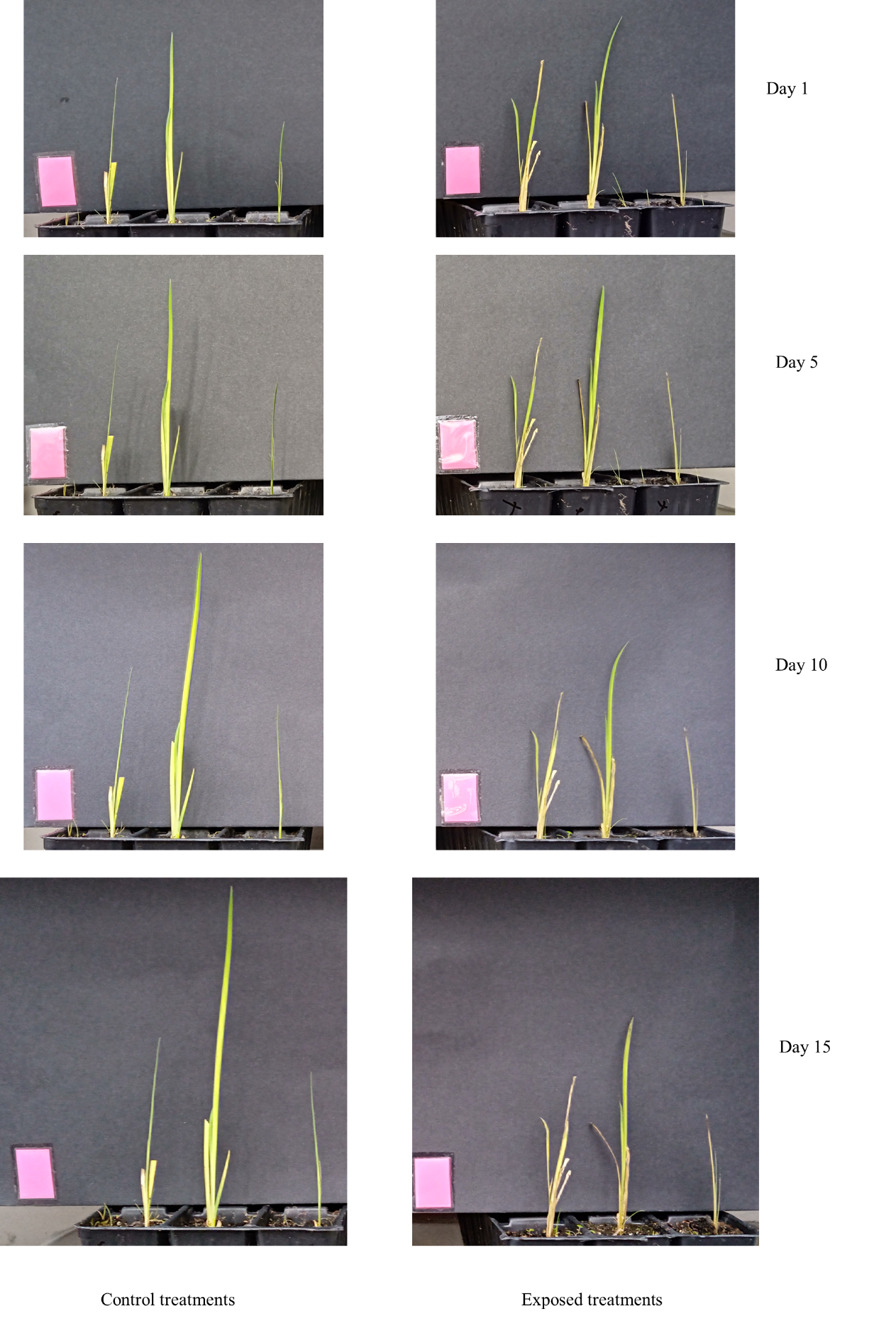


**Supplementry Figure 10.** Changes in growth of *Typha angustifolia* at 1, 5, 10, and 15 days.

**Supplementary Table 2**. Metal(loid)s content recovery certified and obtained values in comparison with standard reference material used.

| Metal(loid)s studied | Certified value* | Obtained value | Recovery | RSD |
| --- | --- | --- | --- | --- |
|  | mg kg^-1^ | mg kg^-1^ | % | % |
| As | 0.042 ±0.010 | 0.04 ±0.001 | 95.32 | 4.95 |
| Cd | 0.120 ±0.007 | 0.116 ±0.003 | 96.94 | 3.18 |
| Cu | 10.200 ±0.500 | 9.949 ±0.226 | 97.54 | 2.56 |
| Ni | 15.200 ±0.600 | 14.517 ±0.486 | 95.5 | 4.78 |
| Pb | 1.670 ±0.110 | 1.621 ±0.045 | 97.07 | 3.08 |
| Zn | 30.500 ±1.100 | 29.945 ±0.412 | 98.18 | 1.87 |
| *Certified values for ERM-CD281 Rye grass certified material | | | | |


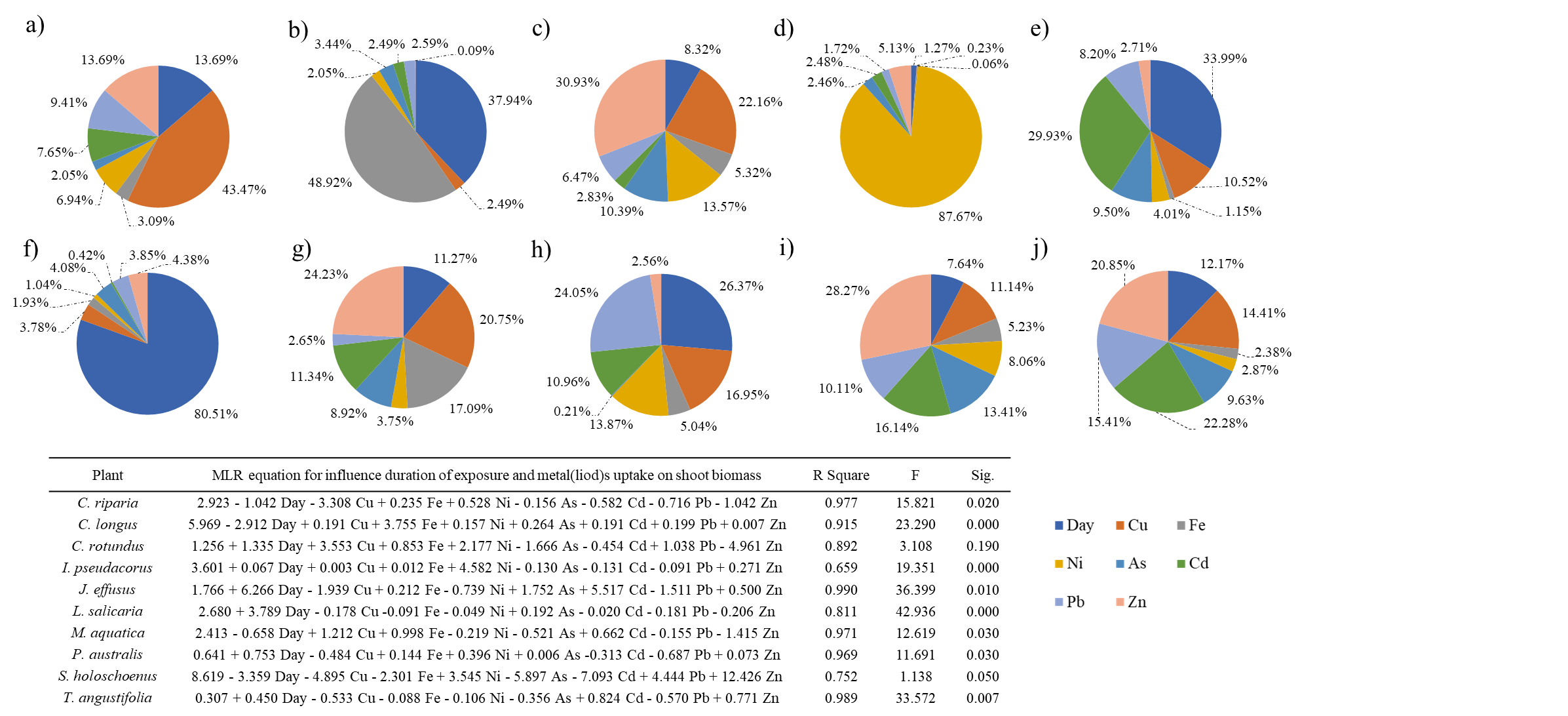


**Supplementry Figure 11.** MLR analysis to assess the effects of exposure time to polluted groundwater and metal(loid) uptake on the shoot biomass production of the studied plants. Pie charts indicate the relative contributions of exposure time and individual metal(loid) concentrations to the total model variability in the following order: a) *C. riparia*, b) *C. longus*, c) *C. rotundus*, d) *I. pseudacorus*, e) *J. effusus*, f) *L. salicaria*, g) *M. aquatica*, h) *P. australis*, i) *S. holoschoenus*, and j) *T. angustifolia.*


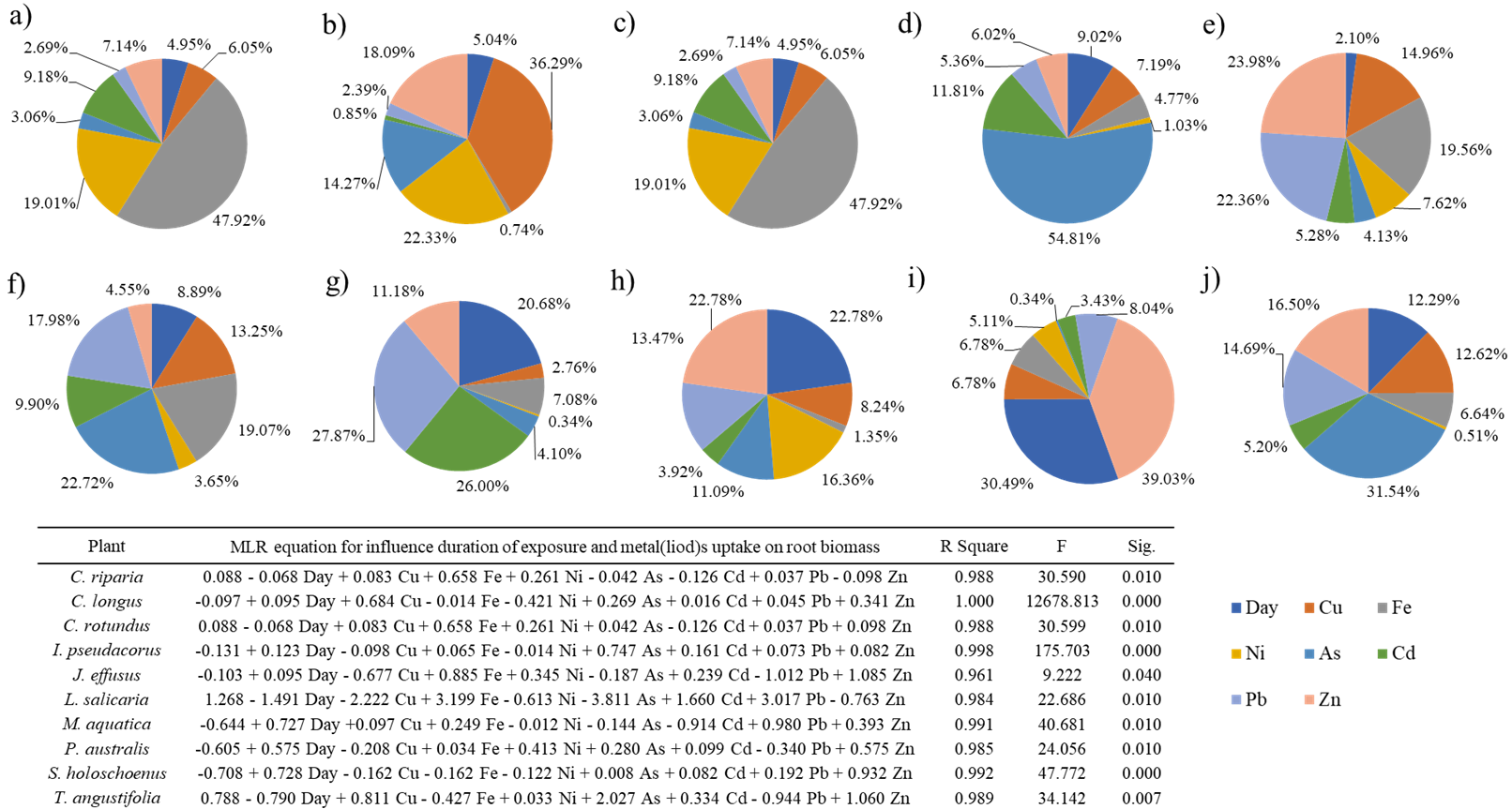


**Supplementry Figure 12.** MLR analysis to assess the effects of exposure time to polluted groundwater and metal(loid) uptake on the root biomass production of the studied plants. Pie charts indicate the relative contributions of exposure time and individual metal(loid) concentrations to the total model variability in the following order: a) *C. riparia*, b) *C. longus*, c) *C. rotundus*, d) *I. pseudacorus*, e) *J. effusus*, f) *L. salicaria*, g) *M. aquatica*, h) *P. australis*, i) *S. holoschoenus*, and j) *T. angustifolia.*

**Supplementary Table 3.** Metal(loid) concentrations in the shoots of the studied aquatic plants grown in polluted groundwater.

| Plant | Days ^+^ | As^1^ | Cd^1^ | Cu^2^ | Fe^2^ | Ni^1^ | Pb^1^ | Zn^2^ |
| --- | --- | --- | --- | --- | --- | --- | --- | --- |
| *Carex riparia* | 5 | 2.96 ±0.11^a^ | 1.06 ±0.03^a^ | 86.08 ±4.49^b^ | 833.38 ±52.7^b^ | 67.44 ±1.86^b^ | 0.38 ±0.04^b^ | 56.48 ±3.76^b^ |
|  | 10 | 3.17 ±0.10^a^ | 1.22 ±0.07^a^ | 116.12 ±8.15^a^ | 859.03 ±8.36^b^ | 217.07 ±15.27^a^ | 0.52 ±0.02^a^ | 74.95 ±2.31^a^ |
|  | 15 | 1.95 ±0.02^b^ | 0.83 ±0.02^b^ | 65.47 ±1.47^b^ | 1138.30 ±113.53^a^ | 62.73 ±1.98^b^ | 0.54 ±0.02^a^ | 56.99 ±4.98^b^ |
| *Cyperus longus* | 5 | 0.23 ±0.01^b^ | 0.81 ±0.10^b^ | 20.93 ±1.15^b^ | 123.25 ±6.53^c^ | 53.16 ±3.77^b^ | 0.38 ±0.02^a^ | 40.16 ±19.70^b^ |
|  | 10 | 0.26 ±0.03^b^ | 1.73 ±0.10^a^ | 90.58 ±3.10^a^ | 272.72 ±9.34^b^ | 118.47 ±4.43^a^ | 0.30 ±0.02^ab^ | 76.77 ±3.52^a^ |
|  | 15 | 0.41 ±0.01^a^ | 1.90 ±0.06^a^ | 102.28 ±6.88^a^ | 399.87 ±9.84^a^ | 72.73 ±3.99^b^ | 0.20 ±0.02^b^ | 60.28 ±2.76^a^ |
| *Cyperus rotundus* | 5 | 1.91 ±0.09^b^ | 0.83 ±0.05^a^ | 65.64 ±0.77^a^ | 937.13 ±29.74^c^ | 70.94 ±6.53^a^ | 0.33 ±0.01^a^ | 46.88 ±2.32^b^ |
|  | 10 | 2.55 ±0.21^ab^ | 0.50 ±0.04^b^ | 66.63 ±2.21^a^ | 2273.51 ±141.77^b^ | 40.26 ±3.04^b^ | 0.52 ±0.03^b^ | 51.19 ±2.00^b^ |
|  | 15 | 3.72 ±0.27^a^ | 0.62 ±0.03^b^ | 69.32 ±3.21^a^ | 3297.38 ±125.00^a*^ | 31.63 ±1.17^b^ | 0.53 ±0.06^b^ | 67.15 ±4.48^a^ |
| *Iris pseudacorus* | 5 | 0.34 ±0.01^a^ | 0.02 ±0.00^a^ | 8.95 ±0.35^b^ | 10.00 ±0.87^b^ | 0.10 ±0.00^b^ | 0.15 ±0.01^a^ | 15.45 ±0.50^b^ |
|  | 10 | 0.35 ±0.03^a^ | 0.02 ±0.00^a^ | 9.04 ±0.59^b^ | 30.01 ±0.02^a^ | 0.11 ±0.01^b^ | 0.17 ±0.00^a^ | 22.18 ±2.26^b^ |
|  | 15 | 0.53 ±0.02^a^ | 0.06 ±0.01^a^ | 12.31 ±1.44^a^ | 41.15 ±0.43^a^ | 6.28 ±1.00^a^ | 0.22 ±0.01^a^ | 39.53 ±1.38^a^ |
| *Juncus effusus* | 5 | 1.44 ±0.03^b^ | 0.73 ±0.18^b^ | 58.39 ±6.08^c^ | 666.79 ±1.56^b^ | 35.03 ±4.08^c^ | 0.24 ±0.01^b^ | 35.08 ±3.05^c^ |
|  | 10 | 2.07 ±0.39^a^ | 1.67 ±0.22^b^ | 153.59 ±6.59^b^ | 1984.45 ±132.12^a^ | 133.32 ±24.69^b^ | 0.38 ±0.01^a^ | 94.73 ±5.52^b^ |
|  | 15 | 2.06 ±0.25^a^ | 3.85 ±0.02^a*^ | 347.07 ±44.63^a*^ | 2082.25 ±65.37^a^ | 313.50 ±2.19^a*^ | 0.41 ±0.01^a^ | 212.27 ±8.02^a*^ |
| *Lythrum salicaria* | 5 | 4.00 ±0.76^a*^ | 0.75 ±0.10^b^ | 81.96 ±3.22^b^ | 551.39 ±6.76^b^ | 16.55 ±1.99^b^ | 0.45 ±0.04^a^ | 92.29 ±1.56^a*^ |
|  | 10 | 1.61 ±0.20^b^ | 0.13 ±0.01^c^ | 84.44 ±3.82^b^ | 987.05 ±68.89^a^ | 19.86 ±0.76^b^ | 0.45 ±0.04^a^ | 96.04 ±2.89^a^ |
|  | 15 | 1.46 ±0.19^b^ | 1.09 ±0.02^a^ | 175.53 ±15.93^a^ | 1284.89 ±28.20^a^ | 131.88 ±8.75^a^ | 0.46 ±0.04^a^ | 103.15 ±10.88^a^ |
| *Mentha aquatica* | 5 | 2.51 ±0.01^b^ | 1.66 ±0.13^a*^ | 58.92 ±3.90^b^ | 1378.92 ±158.95^a*^ | 102.51 ±0.64^b^ | 0.58 ±0.07^a^ | 62.83 ±4.91^b^ |
|  | 10 | 2.66 ±0.09^b^ | 1.42 ±0.21^a^ | 67.05 ±6.89^b^ | 861.05 ±35.20^b^ | 126.46 ±7.88^ab^ | 0.58 ±0.15^a^ | 63.84 ±2.13^b^ |
|  | 15 | 4.66 ±0.01^a^ | 1.43 ±0.05^a^ | 108.95 ±7.53^a^ | 838.90 ±56.40^b^ | 143.72 ±6.02^a^ | 0.65 ±0.02^a^ | 87.42 ±8.52^a^ |
| *Phragmites australis* | 5 | 3.95 ±0.12^b*^ | 0.01 ±0.00^a^ | 49.67 ±4.20^b^ | 831.46 ±30.38^c^ | 7.43 ±0.22^b^ | 0.98 ±0.07^a*^ | 42.38 ±2.61^b^ |
|  | 10 | 4.15 ±0.23^ab*^ | 0.02 ±0.00^a^ | 55.67 ±1.63^ab^ | 1352.89 ±54.94^b^ | 16.76 ±1.82^ab^ | 1.11 ±0.11^a*^ | 62.28 ±0.81^a^ |
|  | 15 | 5.05 ±0.46^a*^ | 0.03 ±0.00^a^ | 62.57 ±4.93^a^ | 2068.19 ±26.61^a^ | 19.92 ±2.62^a^ | 1.48 ±0.38^a*^ | 68.68 ±0.33^a^ |
| *Scirpus holoschoenus* | 5 | 2.06 ±0.08^a^ | 0.47 ±0.04^a^ | 41.51 ±1.47^b^ | 1243.57 ±61.80^b*^ | 23.83 ±4.32^b^ | 0.55 ±0.04^b^ | 48.58 ±2.51^b^ |
|  | 10 | 2.35 ±0.28^a^ | 0.45 ±0.01^a^ | 47.94 ±17.69^b^ | 1443.83 ±299.24^b*^ | 25.67 ±2.90^b^ | 0.68 ±0.00^a^ | 58.81 ±2.23^b^ |
|  | 15 | 2.89 ±0.18^a^ | 0.45 ±0.02^a^ | 69.34 ±7.18^a^ | 2841.99 ±112.62^a*^ | 33.14 ±1.41^a^ | 0.72 ±0.07^a^ | 74.57 ±4.34^a^ |
| *Typha angustifolia* | 5 | 0.22 ±0.01^b^ | 0.66 ±0.01^c^ | 125.40 ±0.96^b*^ | 495.64 ±42.69^c^ | 115.85 ±9.94^b*^ | 1.20 ±0.03^b*^ | 68.32 ±0.57^c^ |
|  | 10 | 1.44 ±0.26^a^ | 6.18 ±0.12^a*^ | 244.36 ±0.42^a*^ | 1132.43 ±54.48^b^ | 426.20 ±12.47^a*^ | 0.69 ±0.03^a^ | 229.19 ±6.34^a*^ |
|  | 15 | 0.65 ±0.00^b^ | 2.95 ±0.19^b^ | 303.09 ±5.43^a*^ | 2760.43 ±188.91^a*^ | 173.90 ±1.21^b^ | 0.57 ±0.01^a^ | 168.76 ±7.35^b^ |
| ^+^Days of Exposure. In super script alphabets indicate significant difference between different exposure duration (5, 10, or 15 days) of the specified aquatic plant’s shoot with “a” being highest followed by later alphabets, while “*” indicate significant highest metal(loid)s’ uptake among different aquatic plant within the same duration of exposure (5, 10, & 15 days).  ^1^The values of As, Cd, Ni, and Pb were presented in μg kg^-1^ of the plant dried biomass.  ^2^For Cu, Fe, and Zn the values were presented in mg kg^-1^ of the plant dried biomass. | | | | | | | | |

**Supplementary Table 4.** Metal(loid) concentrations in the roots of the studied aquatic plants grown in polluted groundwater.

| Plant | Days ^+^ | As^1^ | Cd^1^ | Cu^2^ | Fe^2^ | Ni^1^ | Pb^1^ | Zn^2^ |
| --- | --- | --- | --- | --- | --- | --- | --- | --- |
| *Carex riparia* | 5 | 36.11 ±1.48^b^ | 3.34 ±0.29^b*^ | 809.00 ±12.59^a*^ | 5917.89 ±203.44^c^ | 196.43 ±2.12^b*^ | 0.74 ±0.07^a^ | 150.69 ±3.95^b*^ |
|  | 10 | 47.10 ±0.45^a*^ | 5.31 ±0.30^a*^ | 838.40 ±3.35^a^ | 9988.41 ±177.72^b^ | 305.22 ±11.31^a*^ | 1.10 ±0.08^a^ | 212.69 ±8.41^a*^ |
|  | 15 | 33.61 ±2.40^b^ | 2.79 ±0.13^b^ | 944.63 ±6.22^a^ | 18521.65 ±703.85^a^ | 359.94 ±34.25^a*^ | 1.20 ±0.11^a^ | 218.04 ±10.99^a*^ |
| *Cyperus longus* | 5 | 30.74 ±1.16^b^ | 2.22 ±0.33^b^ | 391.06 ±9.21^b^ | 4055.53 ±203.90^c^ | 142.24 ±7.75^b^ | 0.44 ±0.03^b^ | 90.78 ±1.24^c^ |
|  | 10 | 39.03 ±3.84^b^ | 3.55 ±0.15^ab^ | 761.44 ±7.95^a^ | 8615.95 ±551.33^b^ | 215.33 ±14.46^ab^ | 0.85 ±0.01^a^ | 130.21 ±11.96^b^ |
|  | 15 | 65.95 ±0.33^a*^ | 4.73 ±0.04^a*^ | 822.40 ±29.48^a^ | 21859.01 ±784.53^a*^ | 364.78 ±67.56^a*^ | 0.88 ±0.06^a^ | 232.75 ±12.08^a*^ |
| *Cyperus rotundus* | 5 | 13.09 ±2.16^b^ | 1.53 ±0.06^b^ | 332.04 ±27.11^b^ | 2574.41 ±242.31^b^ | 110.85 ±6.40^b^ | 0.68 ±0.04^b^ | 94.29 ±5.50^a^ |
|  | 10 | 24.25 ±1.46^ab^ | 1.97 ±0.31^ab^ | 363.30 ±30.13^ab^ | 3236.00 ±191.06^ab^ | 130.01 ±5.45^a^ | 1.14 ±0.14^a^ | 82.67 ±1.66^a^ |
|  | 15 | 30.97 ±0.41^a^ | 2.57 ±0.29^a^ | 445.16 ±6.72^a^ | 4877.00 ±118.66^a^ | 146.80 ±5.84^a^ | 1.07 ±0.04^a^ | 99.01 ±25.87^a^ |
| *Iris pseudacorus* | 5 | 2.47 ±0.51^b^ | 0.02 ±0.00^c^ | 15.85 ±6.62^b^ | 243.71 ±75.01^b^ | 2.88 ±0.35^c^ | 0.25 ±0.00^b^ | 14.52 ±6.08^c^ |
|  | 10 | 10.37 ±1.35^a^ | 0.31 ±0.14^b^ | 15.81 ±3.39^a^ | 1185.89 ±671.99^a^ | 47.20 ±0.80^b^ | 0.45 ±0.03^a^ | 53.33 ±3.33^b^ |
|  | 15 | 9.15 ±2.83^a^ | 4.21 ±1.24^a*^ | 30.27 ±4.86^a^ | 1000.72 ±119.73^a^ | 214.90 ±7.04^a^ | 0.40 ±0.04^a^ | 192.90 ±46.56^a^ |
| *Juncus effusus* | 5 | 9.36 ±0.66^b^ | 0.48 ±0.17^c^ | 136.57 ±33.78^b^ | 9757.91 ±3224.46^ab*^ | 78.09 ±5.66 ^a^ | 0.78 ±0.05^b^ | 49.11 ±1.76^b^ |
|  | 10 | 10.06 ±2.41^b^ | 1.29 ±0.31^b^ | 184.05 ±7.22^b^ | 12132.04 ±617.66 ^a*^ | 102.20 ±8.11 ^a^ | 1.24 ±0.05^a^ | 81.29 ±7.63^a^ |
|  | 15 | 29.48 ±0.72^a^ | 2.09 ±0.95^a^ | 494.53 ±9.35^a^ | 8668.55 ±534.60^b^ | 124.22 ±5.26 ^a^ | 1.72 ±0.04^a^ | 95.19 ±3.80^a^ |
| *Lythrum salicaria* | 5 | 1.73 ±0.65^b^ | 0.44 ±0.29^c^ | 45.99 ±19.97^c^ | 1880.43 ±11.80^b^ | 31.71 ±9.40^c^ | 1.31 ±0.05^b^ | 34.71 ±7.50c |
|  | 10 | 6.73 ±1.74^a^ | 1.08 ±0.10^b^ | 220.25 ±9.81^b^ | 4849.68 ±608.88^a^ | 85.84 ±8.00^b^ | 3.95 ±0.85^a^ | 119.36 ±9.01^ab^ |
|  | 15 | 7.39 ±0.90^a^ | 3.12 ±1.56^a^ | 651.28 ±6.47^a^ | 4924.79 ±979.43^a^ | 169.63 ±26.22^a^ | 4.26 ±0.07^a^ | 168.99 ±56.15^a^ |
| *Mentha aquatica* | 5 | 8.15 ±0.81^b^ | 1.55 ±0.11^b^ | 399.20 ±55.39^c^ | 3059.64 ±46.42^c^ | 85.02 ±1.31^b^ | 0.83 ±0.01^c^ | 61.29 ±4.51^b^ |
|  | 10 | 15.87 ±0.69^a^ | 2.05 ±0.18^ab^ | 575.52 ±46.12^b^ | 5034.99 ±481.51^b^ | 76.93 ±1.01^b^ | 1.61 ±0.13^b^ | 66.70 ±2.89^b^ |
|  | 15 | 19.35 ±1.42^a^ | 3.34 ±0.57^a^ | 1199.43 ±27.77^a*^ | 18022.02 ±1508.76^a^ | 266.92 ±13.78^a^ | 3.14 ±0.29^a^ | 131.63 ±13.64^a^ |
| *Phragmites australis* | 5 | 42.30 ±2.92^a*^ | 0.72 ±0.02^c^ | 134.42 ±7.76^b^ | 1935.65 ±124.36^b^ | 32.06 ±0.79^c^ | 0.25 ±0.03^a^ | 46.14 ±4.89^c^ |
|  | 10 | 43.73 ±1.71^a*^ | 2.20 ±0.36^b^ | 213.89 ±1.37^a^ | 4240.53 ±156.04^a^ | 112.13 ±6.40^b^ | 0.72 ±0.03^b^ | 108.19 ±6.96 ^ab^ |
|  | 15 | 31.37 ±2.88^b^ | 5.53 ±0.18^a*^ | 138.04 ±6.08^b^ | 4629.78 ±571.81^a^ | 259.60 ±29.62^a^ | 0.81 ±0.10^b^ | 131.43 ±4.28^a^ |
| *Scirpus holoschoenus* | 5 | 26.02 ±4.65^b^ | 2.13 ±0.40^b^ | 299.50 ±28.45^b^ | 3680.55 ±423.89^c^ | 78.17 ±5.43^c^ | 2.50 ±0.11^c*^ | 103.74 ±1.92^b^ |
|  | 10 | 29.61 ±3.52^b^ | 3.34 ±0.03^a^ | 664.06 ±12.83^a^ | 6988.08 ±299.24^b^ | 259.00 ±3.21^b^ | 4.85 ±0.16^ab*^ | 201.09 ±6.69^a*^ |
|  | 15 | 52.94 ±5.80^a^ | 3.71 ±0.33^a^ | 671.77 ±23.65^a^ | 16029.59 ±1438.71^a^ | 335.66 ±3.49^a^ | 6.12 ±0.47^a*^ | 208.96 ±14.89^a*^ |
| *Typha angustifolia* | 5 | 8.40 ±0.55^c^ | 1.31 ±0.26^b^ | 308.09 ±21.83^c^ | 2427.03 ±206.66^b^ | 86.55 ±3.12^b^ | 0.48 ±0.03^b^ | 79.27 ±3.89^c^ |
|  | 10 | 32.77 ±0.35^a^ | 4.24 ±0.21^a^ | 937.20 ±31.52^a*^ | 4349.55 ±99.48^a^ | 250.13 ±1.18^a^ | 1.69 ±0.08^a^ | 214.64 ±6.33^a*^ |
|  | 15 | 18.15 ±1.69^b^ | 3.09 ±0.19^a^ | 535.39 ±34.04^b^ | 5657.43 ±327.22 ^a^ | 292.95 ±9.03^a^ | 1.88 ±0.11^a^ | 156.09 ±24.25^b^ |
| ^+^Days of Exposure. In super script alphabets indicate significant difference between different exposure duration (5, 10, or 15 days) of the specified aquatic plant’s root with “a” being highest followed by later alphabets, while “*” indicate significant highest metal(loid)s’ uptake among different aquatic plant within the same duration of exposure (5, 10, & 15 days).  ^1^The values of As, Cd, Ni, and Pb were presented in μg kg^-1^ of the plant dried biomass.  ^2^For Cu, Fe, and Zn the values were presented in mg kg^-1^ of the plant dried biomass. | | | | | | | | |
